# *Porphyromonas gingivalis* induces penetration of lipopolysaccharide and peptidoglycan through the gingival epithelium via degradation of junctional adhesion molecule 1

**DOI:** 10.1371/journal.ppat.1008124

**Published:** 2019-11-07

**Authors:** Hiroki Takeuchi, Naoko Sasaki, Shunsuke Yamaga, Masae Kuboniwa, Michiya Matsusaki, Atsuo Amano

**Affiliations:** 1 Department of Preventive Dentistry, Graduate School of Dentistry, Osaka University, Suita-Osaka, Japan; 2 Joint Research Laboratory (TOPPAN) for Advanced Cell Regulatory Chemistry, Graduate School of Engineering, Osaka University, Suita-Osaka, Japan; 3 Department of Applied Chemistry, Graduate School of Engineering, Osaka University, Suita-Osaka, Japan; University of Washington Medical School, UNITED STATES

## Abstract

*Porphyromonas gingivalis* is a major pathogen in severe and chronic manifestations of periodontal disease, which is one of the most common infections of humans. A central feature of *P*. *gingivalis* pathogenicity is dysregulation of innate immunity at the gingival epithelial interface; however, the molecular basis underlying *P*. *gingivalis*–dependent abrogation of epithelial barrier function remains unknown. Gingival epithelial cells express junctional adhesion molecule (JAM1), a tight junction–associated protein, and JAM1 homodimers regulate epithelial barrier function. Here we show that Arg-specific or Lys-specific cysteine proteases (gingipains) secreted by *P*. *gingivalis* can specifically degrade JAM1 at K134 and R234 in gingival epithelial cells, resulting in permeability of the gingival epithelium to 40 kDa dextran, lipopolysaccharide (LPS), and proteoglycan (PGN). A *P*. *gingivalis* strain lacking gingipains was impaired in degradation of JAM1. Knockdown of JAM1 in monolayer cells and a three-dimensional multilayered tissue model also increased permeability to LPS, PGN, and gingipains. Inversely, overexpression of JAM1 in epithelial cells prevented penetration by these agents following *P*. *gingivalis* infection. Our findings strongly suggest that *P*. *gingivali*s gingipains disrupt barrier function of stratified squamous epithelium via degradation of JAM1, allowing bacterial virulence factors to penetrate into subepithelial tissues.

## Author summary

Periodontal diseases, which are among the most common infections of humans, are characterized by gingival inflammation and destruction of the hard and soft tissues that support the tooth, eventually causing tooth loss. *Porphyromonas gingivalis* is a major pathogen in periodontal diseases. Infection of gingival epithelial cells by *P*. *gingivalis* increases epithelial permeability. However, the molecular mechanism and pathological significance of *P*. *gingivalis*–dependent barrier dysfunction in human gingival epithelium remain unknown. In this study, we developed a three-dimensional multilayered tissue model of gingival epithelium infected by *P*. *gingivalis* and used it to monitor penetration of bacterial products derived from *P*. *gingivalis* and other bacteria. We found that *P*. *gingivalis* proteases, called gingipains, have a potent and specific ability to degrade JAM1, which regulates epithelial barrier function. Mechanistically, gingipains degrade mature form of JAM1 on the plasma membrane, increasing penetration of 40 kDa dextran, lipopolysaccharide, peptidoglycan, and gingipains. Our study provides new insights into the etiological role of *P*. *gingivalis*, leading to periodontal destruction.

## Introduction

Periodontal diseases are characterized by destruction of the gingival tissues of the oral cavity including the alveolar bone, leading eventually to exfoliation of the teeth. They are among the most common infectious diseases of humans, and in developed countries over half of the adult population experiences some form of periodontitis [1]. The disease initiates at the epithelial surfaces of the subgingival compartment, and the gingival epithelium plays a central role in responding to microbial infection and orchestrating immune responses [2].

Many mucosal surfaces of humans are colonized by a diverse and abundant microbiota. In most instances the host remains healthy, in large part due to numerous innate and acquired immune mechanisms that limit microbial intrusion and rapidly kill organisms that traverse epithelial barriers. In periodontal tissues, the epithelium of the subgingival compartment plays a central role in orchestration of innate immunity. Lipopolysaccharide (LPS: endotoxins of gram-negative bacteria) and peptidoglycan (PGN: mesh-like patterns outside the plasma membrane of most bacteria) are prototypical classes of pathogen-associated molecular patterns (PAMPs) that are recognized by Toll-like receptors leading to the activation of pro-inflammatory signaling pathways [3]. In simple columnar epithelium of intestinal tracts, once tissue integrity is disrupted by biofilm-derived noxious stimuli, bacterial products such as LPS and PGN invade the deeper tissue, triggering an inflammatory response [4,5]. By contrast, the sulcular epithelium is stratified squamous, and gingival epithelial cells express cell-to-cell molecular adhesion and sealing complexes, including tight junctions [6]. In systemically healthy patients, the number of inflamed periodontal pockets with bleeding on probing and those with suppuration correlated positively with serum LPS concentration [7]. Additionally, plasma LPS levels correlated with multiple clinical parameters of aggressive periodontitis [8]. The higher levels of LPS in the bloodstream in patients with periodontitis is likely due to the transmission of PAMPs from oral bacteria to gingival tissues; to date, however, no molecular analysis has demonstrated that PAMPs penetrate stratified squamous epithelium, such as gingival epithelial tissues, which could be a critical event in periodontal disease pathogenesis.

Periodontal diseases are multispecies infections involving pathogenic communities in which the microbial constituents exhibit polymicrobial synergy. *Porphyromonas gingivalis*, a keystone periodontal pathogen, can increase the pathogenicity of the entire multispecies periodontal community [9]. *P*. *gingivalis* disrupts the barrier function of epithelial cells by degrading cell-to-cell junction complexes [10]. However, the molecular basis underlying *P*. *gingivalis*–dependent barrier dysfunction in human gingival epithelium remains unknown.

*P*. *gingivalis* expresses Arg-specific and Lys-specific cysteine proteases, termed Arg-gingipains (RgpA and RgpB) and Lys-gingipain (Kgp), respectively, which are tightly involved in periodontal disease pathogenicity [11,12]. In the context of cellular adhesion, Rgp isolated from culture supernatant of *P*. *gingivalis* degrades type I and IV collagens [13]. Rgp is also responsible for the degradation of fibronectin and integrin subunits α2, β1, and β3 in human gingival fibroblasts [14]. In addition, Kgp hydrolyses cadherin 1 (CDH1) in Madin-Darby canine kidney (MDCK) cell monolayers [15]. To date, however, the influence of junctional protein(s) degraded by gingipains on the penetration of PAMPs through human gingival epithelium has not been evaluated.

Cell junctions are predominantly responsible for controlling the paracellular barrier of the epithelium [16]. Cell junctions consist of tight junctions that prevent leakage of transported solutes and water and seal the paracellular pathway. Tight junctions are formed by multiprotein complexes including claudin (CLDN), occludin (OCLN), and tight junction protein (TJP). Human gingival epithelial cells express junctional adhesion molecule 1 (JAM1), an immunoglobulin superfamily protein that is also implicated in the regulation of tight junctions [6,17,18]. JAM1 localizes to the vascular endothelium and the mucosal epithelium of numerous organs, as demonstrated by immunohistochemistry and *in situ* hybridization [19]. Monoclonal antibodies against JAM1 inhibit reassembly of tight junctions and block recovery of transepithelial resistance in T84 human colonic adenocarcinoma cells [20]. Knockdown of JAM1 also increases permeability in SK-CO15 human intestinal epithelial cell monolayers [21]. However, the effect of periodontal pathogens on JAM1 in infection-free human gingival epithelium is ethically and technically difficult to analyze.

The *in vitro* construction of living tissue models composed of human cells and extracellular matrices is in the spotlight in the field of tissue engineering. Three-dimensional tissue models of epithelium enable us to utilize any human cell line, control the number of cell layers, and evaluate epithelial barrier function by monitoring fluorescent materials by photometry or microscopy. A recent study proposed the rapid construction of three-dimensional multilayered tissues by the cell-accumulation technique [22], which is potentially useful for reproducing the pathological condition in periodontal diseases.

In this study, we examined the molecular basis of gingival epithelial barrier dysfunction by *P*. *gingivalis* using a newly developed three-dimensional multilayered tissue model (hereafter, 3D-tissue model), and found that gingipain-mediated degradation of JAM1 played an important role in epithelial dysfunction. JAM1 controlled selective permeability dependently on the molecular weight of target molecules. Gingipains were capable of degrading JAM1 at K134 and R234, thereby inducing gingival epithelial permeability and subsequent transmission of LPS and PGN. This work provides insights into process by which the constitutive immune barrier is subverted during periodontal pathogenesis.

## Results

### *P*. *gingivalis* gingipains degrade JAM1 in gingival epithelial cells

**Fig 1:**
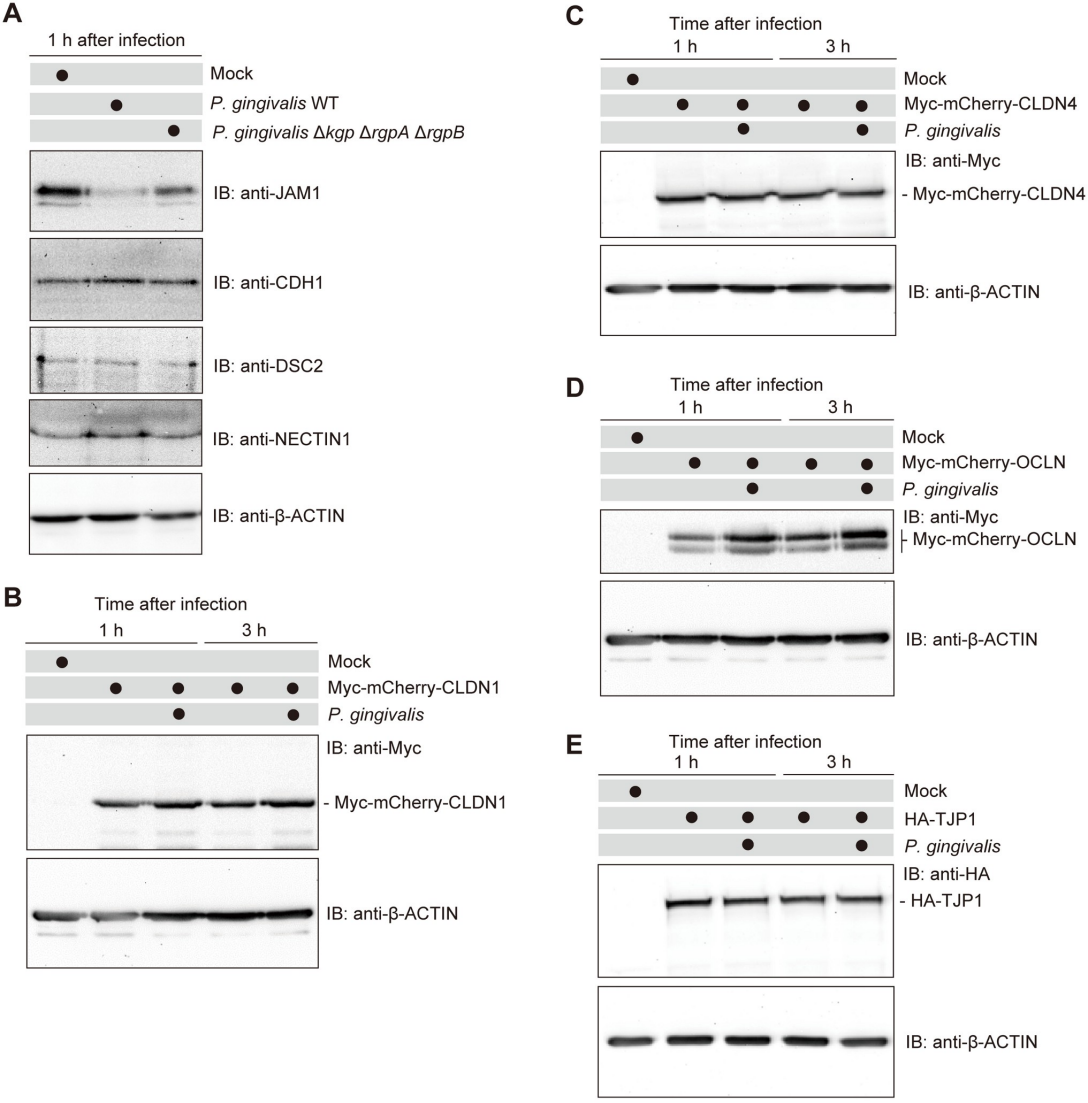
*P*. *gingivalis* gingipains degrade JAM1 in IHGE cells. **(A)** IHGE cells were infected for 1 h with *P*. *gingivalis* WT or the Δ*kgp* Δ*rgpA* Δ*rgpB* mutant at an MOI of 100. The cells were then analyzed by immunoblotting with the indicated antibodies. **(B–E)** IHGE cells were transiently transfected with plasmid encoding Myc-mCherry-CLDN1 (B), Myc-mCherry-CLDN4 (C), Myc-mCherry-OCLN (D), or HA-TJP1 (E). After 48 h of incubation, cells were infected with *P*. *gingivalis* at an MOI of 100 for 1 or 3 h. The cells were then analyzed by immunoblotting with the indicated antibodies. β-actin was used as a loading control. IB, immunoblot.

Previous studies identified the protein components of tight and adherens junctions in human gingival epithelial cells, including JAM1, CDH1, desmocollin 2 (DSC2), nectin cell adhesion molecule 1 (NECTIN1), CLDN, OCLN, and TJP [6]. First, we investigated whether gingipains degrade cell junction molecules at the endogenous protein level. Immortalized human gingival epithelial (IHGE) cells were infected with *P*. *gingivalis* ATCC 33277 wild type (WT) or its isogenic mutant KDP136 (Δ*kgp* Δ*rgpA* Δ*rgpB*) for 1 h at a multiplicity of infection (MOI) of 100. Immunoblots revealed that the levels of JAM1 in IHGE cells were decreased by *P*. *gingivalis* WT, whereas the Δ*kgp* Δ*rgpA* Δ*rgpB* mutant had a negligible effect (Fig 1A). By contrast, *P*. *gingivalis* did not degrade CDH1, DSC2, or NECTIN1 at 1 h after infection. To assess the contribution of secreted gingipains to JAM1 degradation, we collected the bacterial culture supernatant and administered it to IHGE cells for 1 h. Immunoblots revealed that the levels of JAM1 in IHGE cells were decreased by bacterial culture supernatant of *P*. *gingivalis* WT, whereas supernatant from the Δ*kgp* Δ*rgpA* Δ*rgpB* mutant had a negligible effect (S1 Fig). These results suggest that gingipains act specifically on JAM1. To confirm the effects of gingipains on other tight junction proteins, we transfected IHGE cells with plasmid encoding epitope-tagged fusion protein Myc-monomeric Cherry (mCherry), Myc-mCherry-CLDN1, Myc-mCherry-CLDN4, Myc-mCherry-OCLN, or HA-TJP1. Transfected cells were then infected with *P*. *gingivalis* at a MOI of 100 for 1 or 3 h. The protein levels of Myc-mCherry, Myc-mCherry-CLDN1, Myc-mCherry-CLDN4, Myc-mCherry-OCLN, or HA-TJP1 were not decreased by *P*. *gingivalis* infection (Fig 1B–1E and S2 Fig), implying that the bacterium does not degrade the corresponding endogenous proteins.

**Fig 2:**
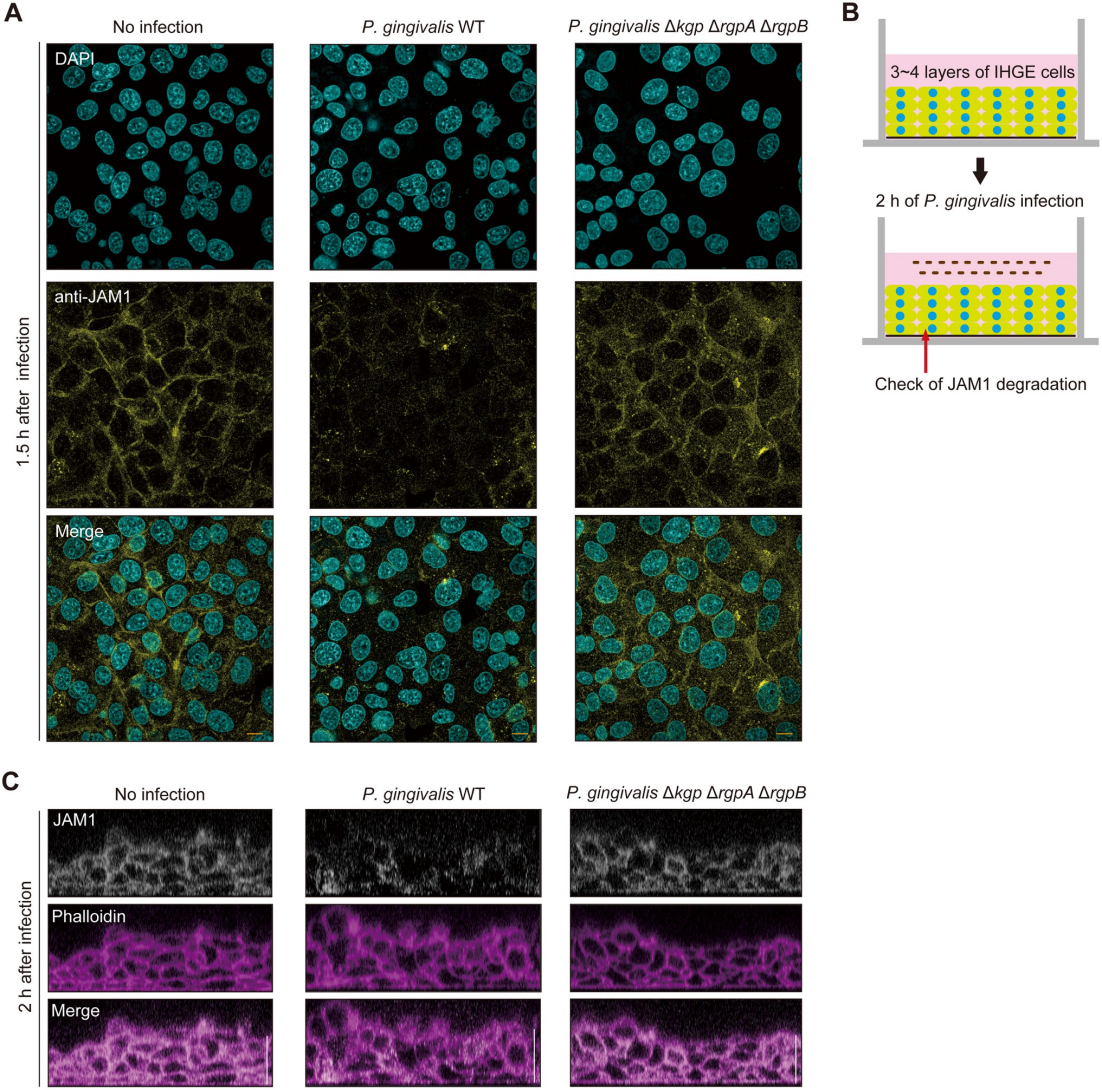
Confocal microscopic images of JAM1 in IHGE cells infected with *P*. *gingivalis* WT or the Δ*kgp* Δ*rgpA* Δ*rgpB* mutant. **(A)** IHGE cells were infected with *P*. *gingivalis* WT or the **Δ***kgp* **Δ***rgpA* **Δ***rgpB* mutant at an MOI of 100 for 1.5 h. The cells were then fixed, stained with DAPI (cyan) and anti-JAM1 (yellow), and analyzed by confocal microscopy. Scale bars, 10 μm. (**B, C)** Schematic illustration (B) and confocal microscopic cross-sectional images (C) of the 3D-tissue model of IHGE cells. Gingival epithelial tissues on coverslips were infected with *P*. *gingivalis* WT or the **Δ***kgp* **Δ***rgpA* **Δ***rgpB* mutant for 2 h. The tissues were then fixed, stained with anti-JAM1 (white) and Alexa Fluor 568–conjugated phalloidin (magenta), and analyzed by confocal microscopy. Scale bars, 30 μm.

Next, we visualized the localization of JAM1 in IHGE cells. The signal intensity of JAM1 was reduced in the peripheral area of IHGE cells infected with *P*. *gingivalis* WT or treated with bacterial culture media of *P*. *gingivalis* WT, 1.5 h after infection or administration (Fig 2A, S3A Fig). By contrast, the Δ*kgp* Δ*rgpA* Δ*rgpB* mutant exerted negligible effects, consistent with the results in Fig 1A and S1 Fig. To assess the contribution of gingipains to JAM1 degradation in deeper epithelium, we employed a newly developed 3D-tissue model of IHGE cells generated by the cell-accumulation technique (Fig 2B and S3B and S4 Figs) [22]. The cell-accumulation technique had a negligible effect on *JAM1* mRNA expression in IHGE cells (S5 Fig). We infected the 3D tissue models with *P*. *gingivalis* WT or Δ*kgp* Δ*rgpA* Δ*rgpB*, or treated them with bacterial culture supernatant from these strains, and then analyzed the degradation of deeply seated JAM1 using confocal microscopy. As shown in Fig 2C and S3C Fig, *P*. *gingivalis* WT caused JAM1 to disappear even in tissues 3–4 layers below the surface by 2 h after infection or administration, whereas the Δ*kgp* Δ*rgpA* Δ*rgpB* mutant did not. These results clearly show that gingipains deeply invade tissue and degrade JAM1 in human gingival epithelial tissues.

### JAM1 is localized in the plasma membrane following cleavage of N-terminal signal peptide

**Fig 3:**
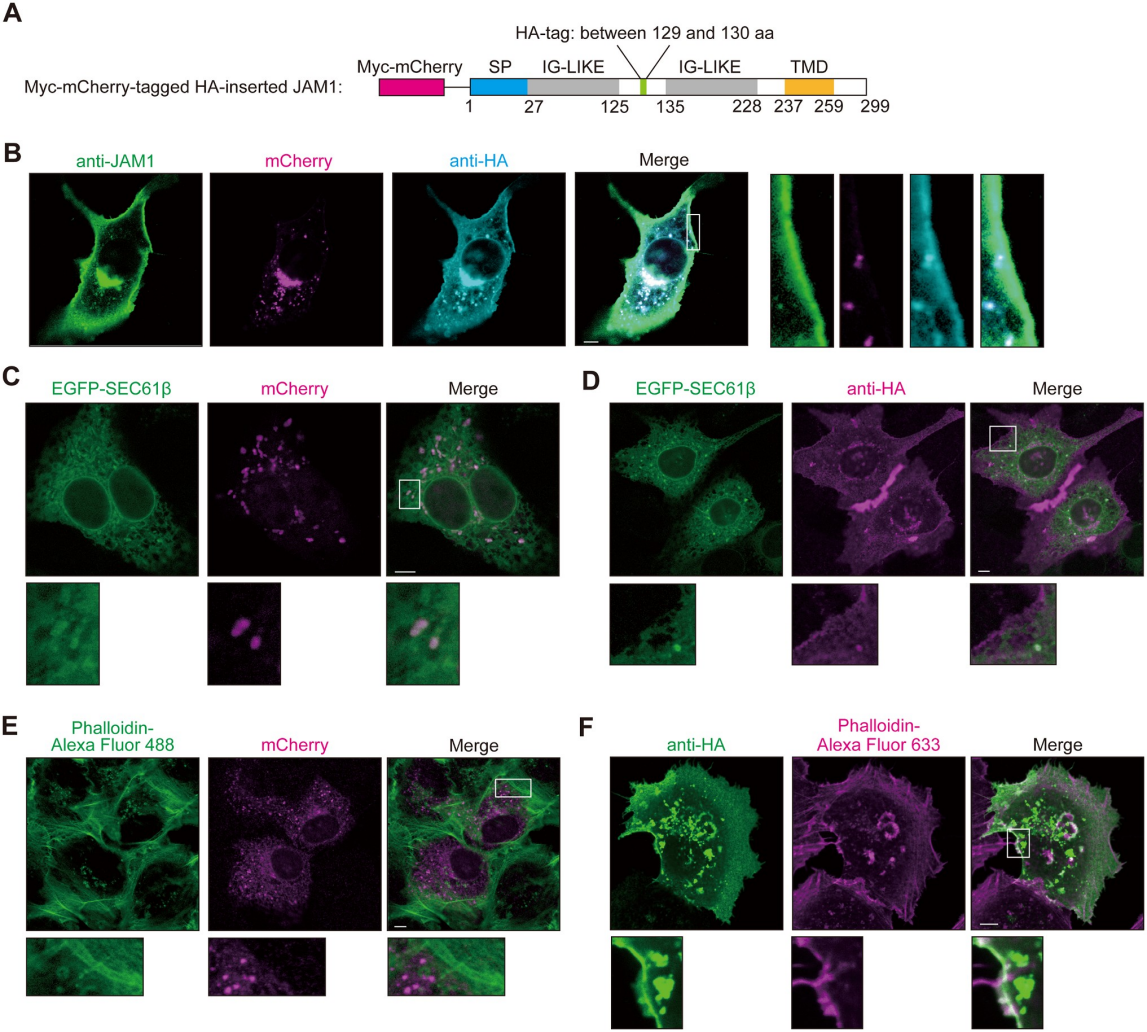
Confocal microscopic images of JAM1 in IHGE cells. **(A)** Schematic view of the JAM1 construct. The Myc-mCherry tag is indicated by the magenta box, and the HA-tag is indicated by the green box. The signal peptide is indicated by the blue box, and IG-LIKE and TMD domains are indicated by the gray boxes and orange box, respectively. **(B)** Confocal microscopic images of an IHGE cell expressing Myc-mCherry-JAM1, stained with anti-JAM1 (green) and anti-HA (cyan). mCherry is shown in magenta. Higher magnifications of the areas indicated by the white boxes in the left panels are shown at right. Scale bar, 5 μm. **(C**–**F)** IHGE cells were transiently transfected with plasmids encoding Myc-mCherry–tagged HA-inserted JAM1; in (C) and (D), the cells were also transfected with EGFP-SEC61β (green). Following 48 h of incubation, cells were fixed, stained with anti-HA (D, magenta; F, green), and also stained with Alexa Fluor 488–conjugated phalloidin (E, green) or Alexa Fluor 633–conjugated phalloidin (F, magenta). The cells were then analyzed by immunofluorescence microscopy. Higher magnifications of the areas indicated by white boxes in the upper panels are shown on the lower side. Scale bars, 5 μm. See also S9A–S9D Fig.

In order to be degraded by gingipains, JAM1 must be localized on the plasma membrane. Hence, we monitored the intracellular localization of JAM1 before and after cleavage of its signal peptide. JAM1 has a putative signal peptide followed by two C2-type immunoglobulin (Ig) domains and a transmembrane domain [23]. We constructed Myc-mCherry–tagged human influenza hemagglutinin (HA)-inserted JAM1 (Fig 3A) and transfected IHGE cells with the vector to determine the protein’s localization by confocal microscopy. As shown in Fig 3B and S6 Fig, the mCherry signal indicating that the cleaved N-terminal region was located in intracellular space, and the anti-HA signal corresponding to the cleaved C-terminal region, were located at the plasma membrane, as well as within the intracellular space. To assess the influence of inserting the Myc-mCherry protein in front of the signal peptide on JAM1 maturation, we introduced a Myc-mCherry–tagged HA-inserted JAM1 plasmid encoding a deletion mutant of the JAM1 signal peptide into IHGE cells (S7A Fig). Immunoblots confirmed that HA-inserted JAM1 was processed in JAM1 WT and Δ (1–16), but not in the Δ (1–17) mutant (S7B Fig). These results indicated that Myc-mCherry–tagged HA-inserted JAM1 was properly processed at the signal peptide. In addition, we stained IHGE cells expressing HA-EGFP (S8A Fig) or Myc-mCherry–tagged HA-inserted JAM1 (S8B Fig) using anti-HA antibody, with or without permeabilization, to confirm that JAM1 was localized on the surface of IHGE cells. HA-EGFP protein in IHGE cells was labeled with anti-HA antibody only in permeabilized cells, indicating that the anti-HA antibody is not capable of staining a cytosolic protein. By contrast, HA-inserted JAM1 protein was labeled with anti-HA antibody even without permeabilization, indicating that HA-inserted JAM1 is on the cell surface. Together, these observations confirmed that HA-inserted JAM1 was properly transported to cell surface.

We next transfected IHGE cells with a plasmid encoding HA-inserted JAM1, and either enhanced green fluorescent protein (EGFP)-SEC61β (marker for endoplasmic reticulum membrane protein) or EGFP-TOMM20 (marker for outer mitochondrial membrane protein; not included in endomembrane system), and determined the co-localization of HA-inserted JAM1 and the organelle marker by confocal microscopy. The mCherry and anti-HA signals co-localized with SEC61β (Fig 3C, 3D and S9A–S9F Fig), but not TOMM20 (S10A–S10D Fig), suggesting that JAM1 transport is mediated by an endomembrane system. Notably, actin stained with phalloidin co-localized with anti–HA-labeled JAM1, but not the mCherry signal (Fig 3E, 3F and S11A and S11B Fig). These results illustrate that JAM1 is transported from the endoplasmic reticulum to the plasma membrane following the cleavage of its signal peptide, and that mature JAM1 at cell surface is a potential target of gingipains.

### *P*. *gingivalis*, but not *S*. *gordonii* or *F*. *nucleatum*, degrades JAM1

**Fig 4:**
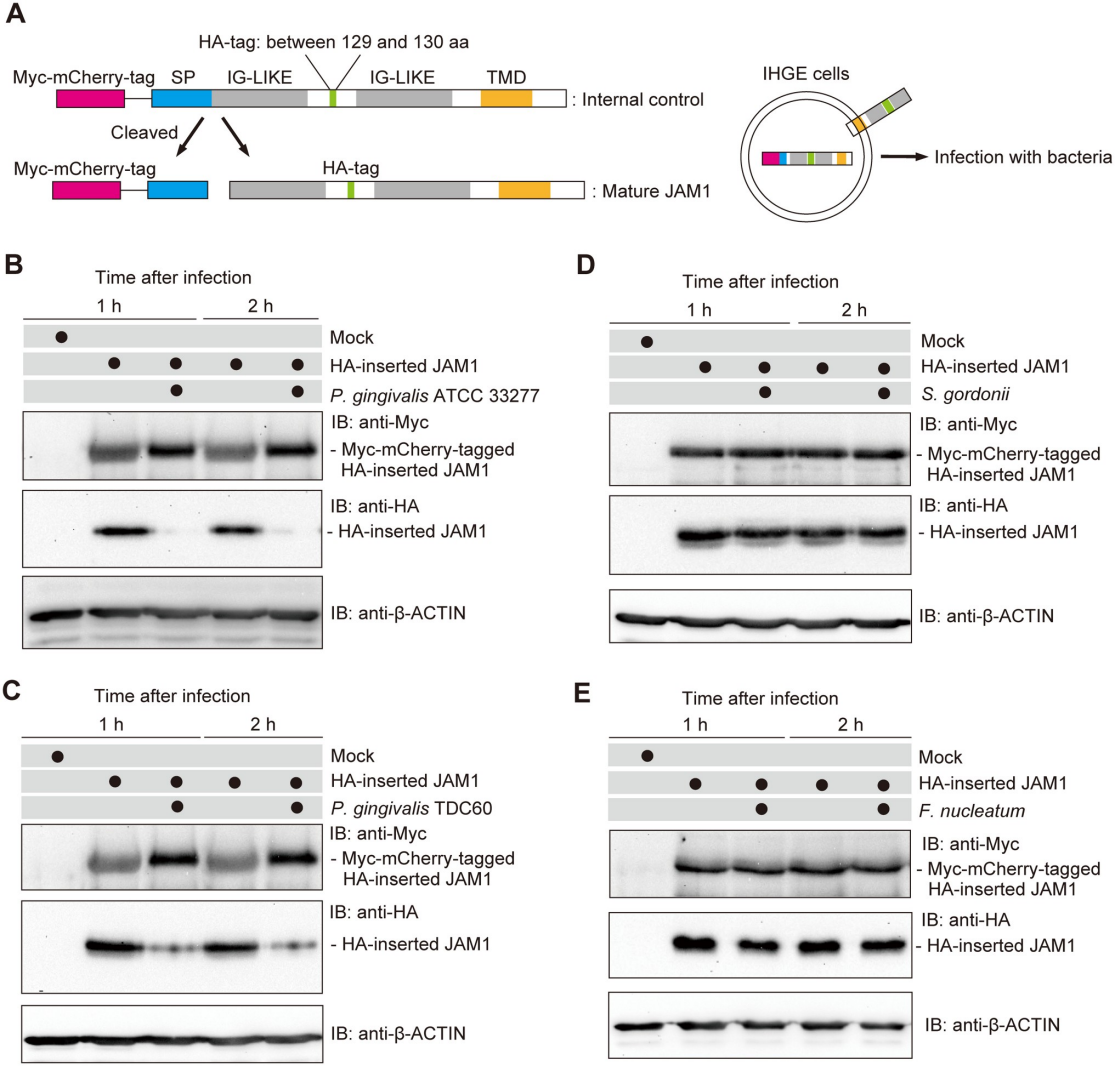
*P*. *gingivalis*, but not *S*. *gordonii* or *F*. *nucleatum*, degrades JAM1 in IHGE cells. **(A)** Schematic view of the structure of Myc-mCherry–tagged HA-inserted JAM1. The immature form of Myc-mCherry–tagged HA-inserted JAM1 was used as an internal control to monitor degradation flux of the mature form of HA-inserted JAM1. IHGE cells were transiently transfected with Myc-mCherry–tagged HA-inserted JAM1 plasmid. Following 48 h of incubation, cells were infected for 1 or 2 h with the indicated bacteria at an MOI of 100. The cells were then analyzed by immunoblotting using the indicated antibodies. **(B**–**E)** IHGE cells were transiently transfected with the HA-inserted JAM1 plasmid. Following 48 h of incubation, the cells were infected with *P*. *gingivalis* ATCC 33277 (B), *P*. *gingivalis* TDC60 (C), *S*. *gordonii* DL-1 (D), or *F*. *nucleatum* ATCC 25586 (E) at an MOI of 100 for the indicated times. The cells were then analyzed by immunoblotting with the indicated antibodies.

The localization of JAM1 in IHGE cells prompted us to examine the degradation of immature and mature JAM1 by *P*. *gingivalis*. Myc-mCherry–tagged HA-inserted JAM1 was ectopically expressed in IHGE cells, and the kinetics of Myc- and HA-tagged JAM1 were examined after infection with *P*. *gingivalis* (Fig 4A). Given the substrate specificity of gingipains, we used the HA tag as a marker of mature JAM1 because its amino acid sequence (YPYDVPDYA) does not contain K or R residues. The N-terminal Myc-mCherry tag was also useful for distinguishing immature and mature JAM1 by molecular weight in immunoblots. As shown in Fig 4B, *P*. *gingivalis* infection decreased the amount of mature JAM1 labeled with anti-HA, but had a negligible effect on the level of immature JAM1 labeled with anti-Myc. Thus, *P*. *gingivalis* targets mature JAM1, but not the immature form. In addition, we infected IHGE cells with *P*. *gingivalis* TDC60 isolated from a severe periodontal lesion [24]. One and two hours after infection, mature JAM1 was markedly less abundant in cells infected with *P*. *gingivalis* TDC60 (Fig 4C), suggesting that JAM1 degradation can also be caused by other *P*. *gingivalis* strains. Furthermore, administration of bacterial culture supernatant of *P*. *gingivalis* WT, but not that of Δ*kgp* Δ*rgpA* Δ*rgpB*, decreased the amount of mature JAM1 (S12 Fig), indicating that secreted gingipains can degrade mature JAM1.

Human oral bacteria including *Fusobacterium nucleatum* and *Streptococcus gordonii* are capable of interacting with their environment by attaching to host surfaces and establishing mixed-species communities [25]. Hence, we infected IHGE cells expressing Myc-mCherry–tagged HA-inserted JAM1 with *S*. *gordonii* or *F*. *nucleatum*. Upon infection with these species, the level of HA-inserted JAM1 was not reduced 2 h after infection (Fig 4D and 4E), suggesting that *S*. *gordonii* and *F*. *nucleatum* are not capable of degrading JAM1.

### JAM1 K134 and R234 are responsible for degradation by *P*. *gingivalis*

**Fig 5:**
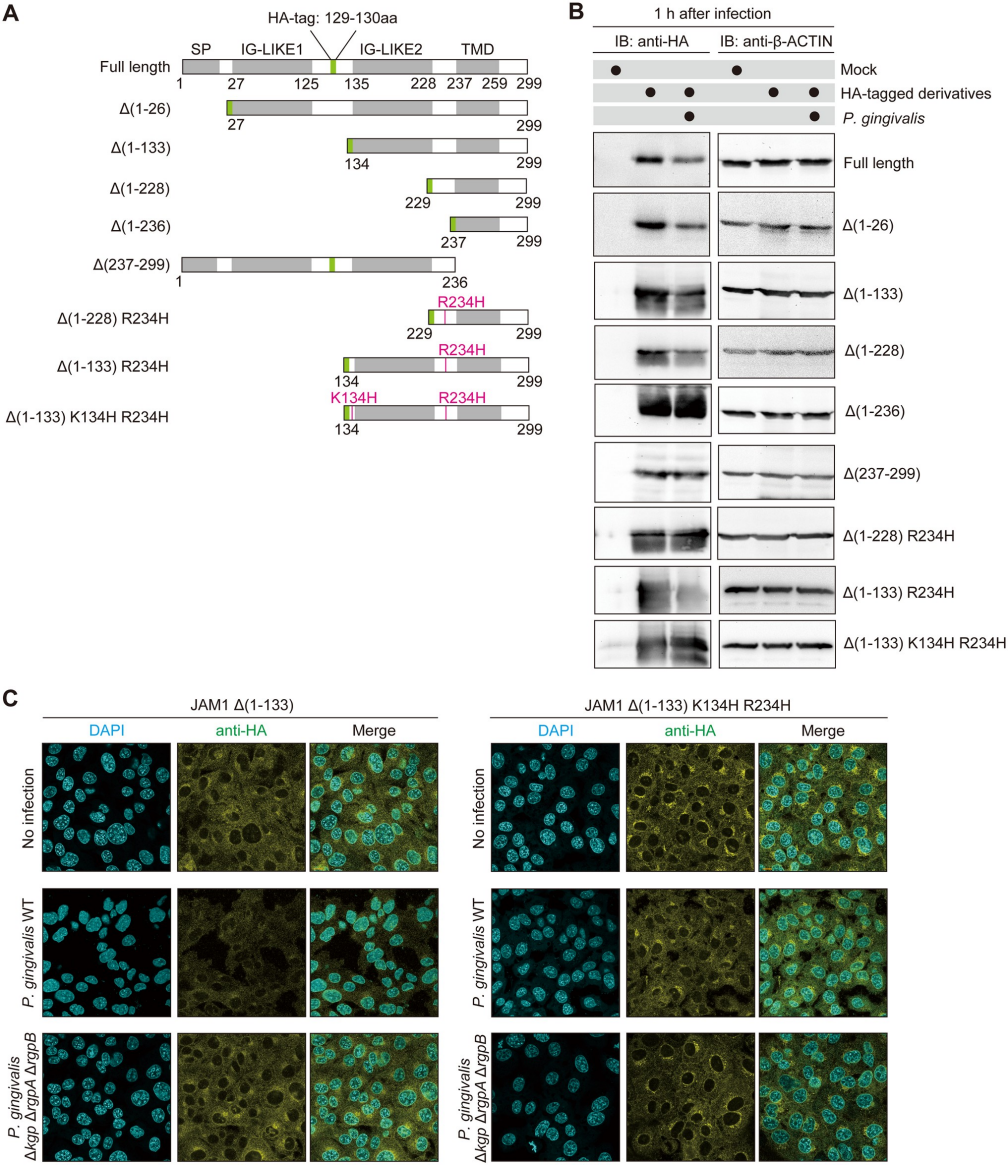
The K134 and R234 residues are involved in degradation of JAM1 by *P*. *gingivalis* in IHGE cells. **(A)** Schematic view of the JAM1 structure and derivatives. SP, IG-LIKE, or TMD domains are indicated by gray boxes. HA-tag is shown in green. The point mutations K134H and R234H are shown in magenta. **(B)** IHGE cells were transiently transfected with plasmid encoding HA-inserted JAM1 or the indicated JAM1 mutants. Following 48 h of incubation, the cells were infected with *P*. *gingivalis* at an MOI of 100 for 1 h, and then analyzed by immunoblotting using the indicated antibodies. **(C)** IHGE cells stably expressing HA-tagged JAM1 Δ (1–133) or HA-tagged JAM1 Δ (1–133) K134H R234H were infected for 1 h with *P*. *gingivalis* WT or the Δ*kgp* Δ*rgpA* Δ*rgpB* mutant at an MOI of 100. The cells were then fixed, stained with DAPI (cyan) and anti-HA (yellow), and analyzed by immunofluorescence microscopy. Scale bars, 10 μm.

To identify the residue(s) responsible for degradation by *P*. *gingivalis* gingipains, we performed a structural analysis of JAM1 using deletion and mutated constructs (Fig 5A). These constructs were expressed in IHGE cells, which were then infected with *P*. *gingivalis*. As shown in Fig 5B, degradation was observed in JAM1 full length, JAM1 Δ (1–26), JAM1 Δ(1–133), and JAM1 Δ(1–228), but not JAM1 Δ(1–236), indicating that the extracellular domain of JAM1 is responsible for bacterial degradation. By contrast, HA-tagged JAM1 Δ (237–299) lacking the transmembrane domain was not decreased by *P*. *gingivalis*, suggesting that membrane localization of JAM1 is needed for bacterial degradation. To detect the residue(s) responsible for degradation of JAM1 by gingipains, we next replaced the R and K residues with H, a basic amino acid, in point mutation constructs. The level of HA-tagged JAM1 Δ (1–228) R234H was not decreased by the infection. Furthermore, the level of HA-tagged JAM1 Δ (1–133) R234H was reduced following infection with *P*. *gingivalis*, but JAM1 Δ (1–133) K134H R234H was not. These results illustrate that K134 and R234 are the residues targeted for degradation by gingipains.

To test the notion that K134 and R234 are the key residues for JAM1 degradation, we performed immunofluorescence assays in IHGE cells expressing HA-tagged JAM1 Δ (1–133) or HA-tagged JAM1 Δ (1–133) K134H R234H following infection with *P*. *gingivalis*. One hour after infection, in IHGE cells expressing HA-tagged JAM1 Δ (1–133) the anti-HA signal was decreased by *P*. *gingivalis* WT, but not by the Δ*kgp* Δ*rgpA* Δ*rgpB* mutant (Fig 5C). By contrast, in cells expressing HA-tagged JAM1 Δ (1–133) K134H R234H, the anti-HA signal was not decreased by either *P*. *gingivalis* WT or Δ*kgp* Δ*rgpA* Δ*rgpB*. These results are consistent with the semiquantitative data in Fig 5B.

### JAM1 prevents penetration of LPS and PGN through gingival epithelium

**Fig 6:**
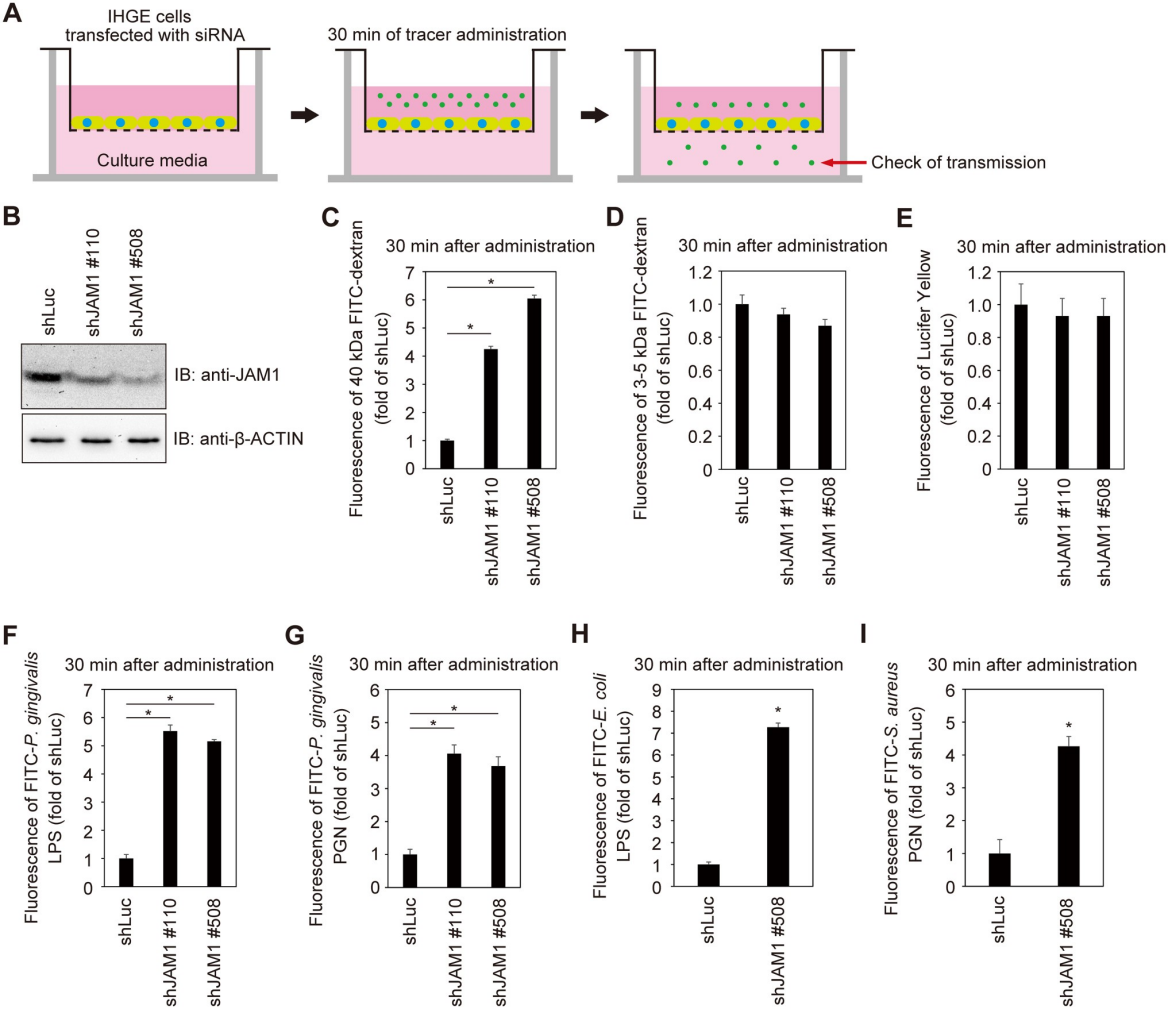
JAM1 is required for epithelial barrier function of IHGE cells. **(A)** Schematic image of the culture insert system. Monolayer of IHGE cells stably expressing shLuc, shJAM1 #110, or shJAM1 #508 were cultured in culture inserts. FITC-labeled tracer was added to culture media in the upper compartment. Following 30 min of incubation, the transmission of tracer from the upper compartment to the lower compartment was analyzed by spectrometry. **(B)** JAM1 expression in IHGE cells stably expressing shLuc, shJAM1 #110, or shJAM1 #508 was analyzed by immunoblotting with the indicated antibodies. **(C**–**I)** Permeability to 40 kDa FITC-dextran (C), 3–5 kDa FITC-dextran (D), 0.5 kDa Lucifer Yellow (E), FITC–*P*. *gingivalis* LPS (F), FITC–*P*. *gingivalis* PGN (G), FITC–*E*. *coli* LPS (H), or FITC–*S*. *aureus* PGN (I) in IHGE cells expressing shLuc and shJAM1. Results are expressed as fold change relative to cells expressing shLuc and are the means ± SD of eight technical replicates. *, p<0.05, two-tailed Dunnett’s test (C-G) or two-tailed *t* test (H, I).

Knockdown of JAM1 disrupts epithelial barrier function in SK-CO15 cells (human intestinal epithelial cell line) [21]. The intestinal epithelium is simple columnar and forms the luminal surface of the small and large intestine of the gastrointestinal tract. By contrast, the gingival epithelium located at the inner edge of the gingival sulcus is stratified squamous, prompting us to clarify the phenotype of JAM1 depletion in gingival epithelium. To assess the contribution of JAM1 expression to the permeability of gingival epithelial cells, we generated IHGE cell lines stably expressing small hairpin RNA (shRNA) against JAM1 (shJAM1 #110 and shJAM1 #508), and then performed permeability assays using a small-molecule fluorescent probe (Fig 6A). Knockdown of JAM1 in each cell line was confirmed by immunoblot (Fig 6B). In addition, by confocal microscopy (S13A Fig) and spectrometry (S13B Fig), we confirmed that 40 kDa fluorescein isothiocyanate (FITC)-dextran was not internalized in IHGE cells 30 min after administration. As shown in Fig 6C, IHGE monolayers expressing shRNA against JAM1 were significantly more permeable to 40 kDa FITC-dextran than control cells expressing shRNA against firefly luciferase (shLuc). By contrast, the permeability to 3–5 kDa FITC-dextran (Fig 6D) and 0.5 kDa Lucifer Yellow (Fig 6E) were not affected by knockdown of JAM1. These results suggest that JAM1 is involved in the kinetics of flux of molecules larger than 5 kDa between gingival epithelial cell layers.

*P*. *gingivalis* expresses danger signals such as LPS and PGN, as well as gingipains. LPS is heterogeneous and tends to form aggregates of varying sizes, but the molecular weight of dissociated LPS is about 20 kDa [26]. PGN is formed from linear chains of two amino sugars, N-acetylglucosamine (GlcNAc) and N-acetylmuramic acid (MurNAc); the molecular weight of PGN monomer (GlcNAc-MurNAc) with cross-linked peptide structures is roughly 40 kDa. Therefore, we hypothesized that knockdown of JAM1 would allow permeation of LPS and PGN. To generate dissociated LPS and PGN, we treated LPS with polysorbate 20 and sodium citrate, and PGN with saliva lysozyme, which can split the peptidoglycan between GlcNAc and MurNAc. We then performed permeability assays with FITC-labeled LPS and PGN. Thirty minutes after administration, permeability to FITC-*P*. *gingivalis* LPS (Fig 6F) and FITC-*P*. *gingivalis* PGN (Fig 6G) was significantly increased by knockdown of JAM1. To confirm the generality of transmission of LPS and PGN following knockdown of JAM1, we also examined the permeability of *Escherichia coli* LPS and *Staphylococcus aureus* PGN through IHGE cells. In cells expressing shJAM1, permeability to FITC–*E*. *coli* LPS (Fig 6H) and FITC–*S*. *aureus* PGN (Fig 6I) was also significantly increased 30 min after administration, suggesting that JAM is involved in the permeability of gingival epithelial cells to LPS and PGN.

**Fig 7:**
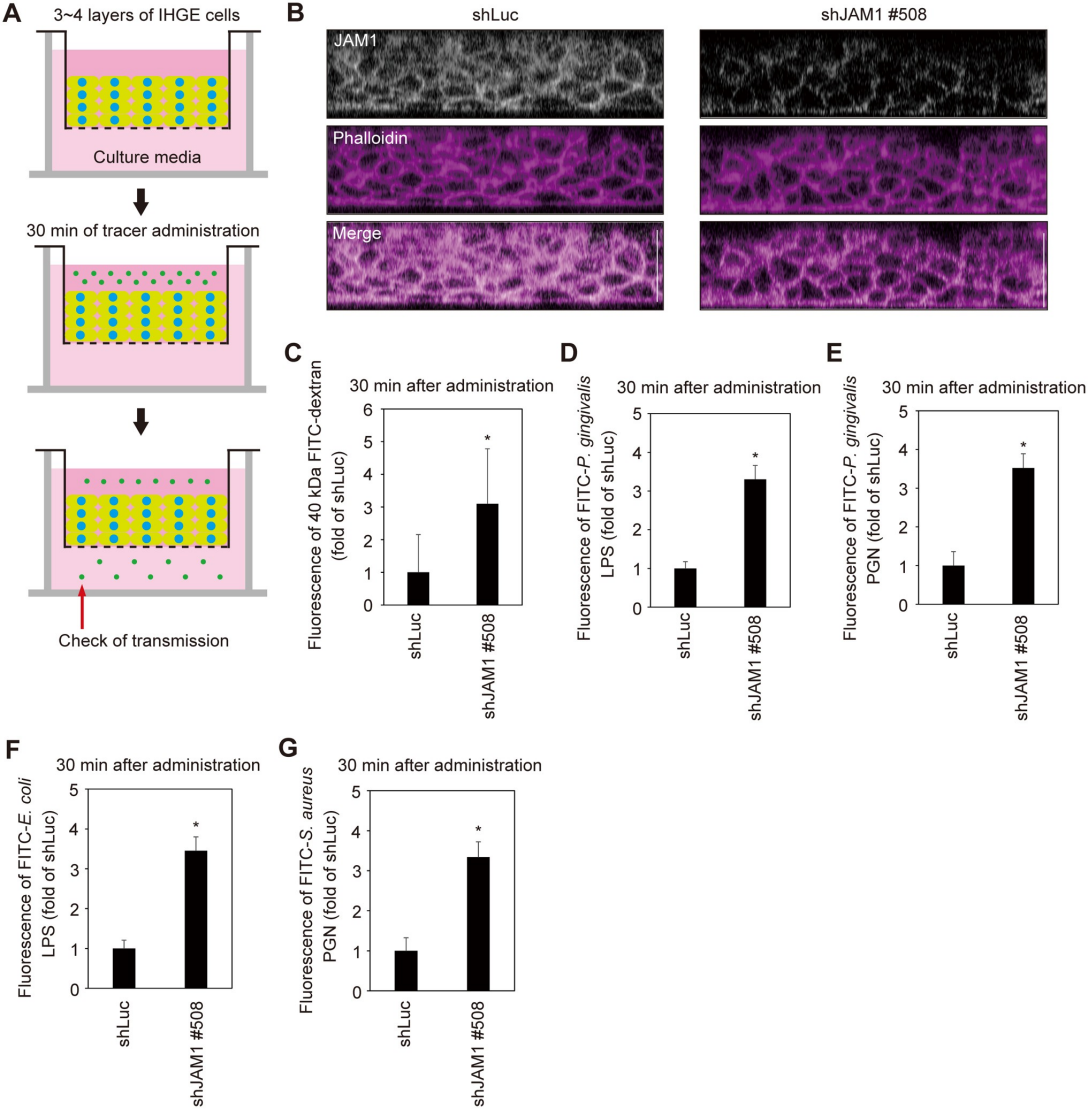
JAM1 is required for epithelial barrier function of gingival epithelial tissues. **(A, B)** Schematic illustration (A) and confocal microscopic cross-sectional images (B) of 3D-tissue model expressing shLuc or shJAM1. Gingival epithelial tissues were fixed, stained with anti-JAM1 (white) and Alexa Fluor 568–conjugated phalloidin (magenta), and analyzed by confocal microscopy. Scale bars, 30 μm. **(C**–**G)** Permeability to 40 kDa FITC–dextran (C), FITC–*P*. *gingivalis* LPS (D), FITC–*P*. *gingivalis* PGN (E), FITC–*E*. *coli* LPS (F), and FITC–*S*. *aureus* PGN (G) in gingival epithelial tissues expressing shLuc and shJAM1. Results are expressed as fold change relative to epithelium expressing shLuc and are the means ± SD of seven technical replicates. *, p<0.05, one-tailed *t* test (C–E).

To assess the contribution of JAM1 to the permeability of deeper epithelium, we generated a 3D-tissue model stably expressing shLuc or shJAM1 using the cell accumulation technique (Fig 7A). Effective suppression of JAM1 expression was confirmed by confocal microscopy (Fig 7B). The tissues were then treated with 40 kDa FITC-dextran, FITC–*P*. *gingivalis* LPS, or FITC–*P*. *gingivalis* PGN, and subjected to permeability assays. Thirty minutes after administration, the permeability to 40 kDa FITC-dextran (Fig 7C), FITC–*P*. *gingivalis* LPS (Fig 7D), FITC–*P*. *gingivalis* PGN (Fig 7E), FITC–*E*. *coli* LPS (Fig 7F), and FITC–*S*. *aureus* PGN (Fig 7G) were significantly increased by knockdown of JAM1 in gingival epithelial tissues. These results suggest that JAM1 impacts the permeability of gingival epithelium to LPS and PGN.

### JAM1 degradation is involved in penetration of gingipains through gingival epithelial barrier

**Fig 8:**
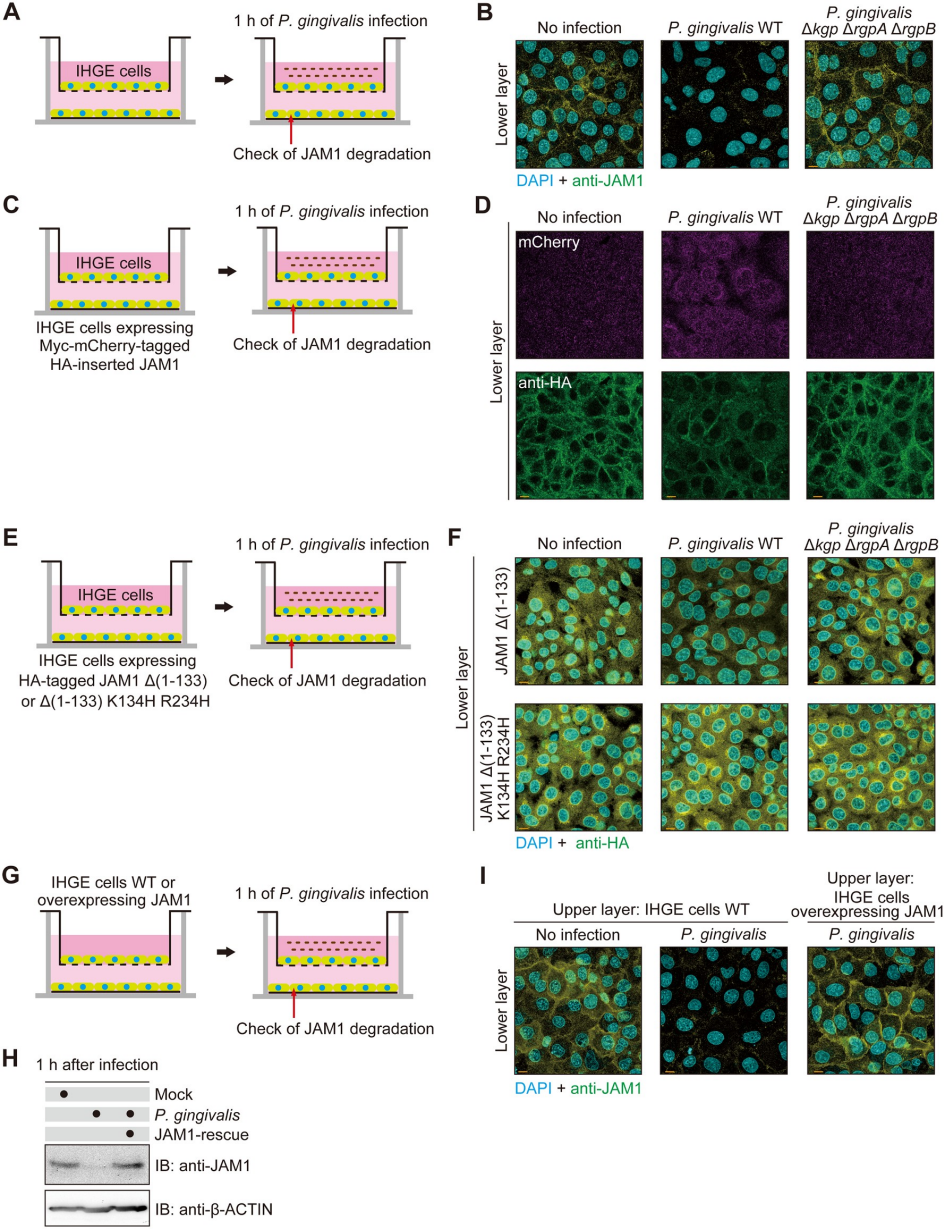
*P*. *gingivalis* gingipains penetrate the epithelial barrier of IHGE cells. **(A, B)** Schematic image of the culture insert system (A). A monolayer of IHGE cells was cultured in the upper compartment and on a coverslip in the lower compartment. Cells in the upper compartment were infected with *P*. *gingivalis* WT or the Δ*kgp* Δ*rgpA* Δ*rgpB* mutant at an MOI of 100. Following 1 h of incubation, cells in the lower compartment were fixed, stained with DAPI (cyan) and anti-JAM1 (yellow), and analyzed by confocal microscopy (B). Scale bars, 10 μm. **(C, D)** Schematic image of the culture insert system (C). A monolayer of IHGE cells (WT) was cultured in the upper compartment and IHGE cells stably expressing Myc-mCherry–tagged HA-inserted JAM1 were cultured on a coverslip in the lower compartment. Cells in the upper compartment were infected with *P*. *gingivalis* WT or the Δ*kgp* Δ*rgpA* Δ*rgpB* mutant at an MOI of 100. Following 1 h of incubation, cells in the lower compartment were fixed, stained with anti-HA (green), and analyzed by confocal microscopy (D). Scale bars, 10 μm. **(E, F)** Schematic image of the culture insert system (E). A monolayer of IHGE cells (WT) was cultured in the upper compartment and IHGE cells stably expressing HA-JAM1 Δ (1–133) or Δ (1–133) K134H R234H were cultured on a coverslip in the lower compartment. Cells in the upper compartment were infected with *P*. *gingivalis* WT or the Δ*kgp* Δ*rgpA* Δ*rgpB* mutant at an MOI of 100. Following 1 h of incubation, cells in the lower compartment were fixed, stained with DAPI (cyan) and anti-HA (yellow), and analyzed by confocal microscopy (F). Scale bars, 10 μm. **(G-I)** Schematic image of the culture insert system (G). JAM1-expressing IHGE cells (WT or overexpressing JAM) were infected with *P*. *gingivalis* for 1 h and analyzed by immunoblotting with the indicated antibodies (H). A monolayer of IHGE cells (WT or overexpressing JAM1) was cultured in the culture insert. Cells were infected with *P*. *gingivalis* at an MOI of 100 in the upper compartment. Following 1 h of incubation, cells in the lower compartment were fixed, stained with DAPI (cyan) and anti-JAM1 (yellow), and analyzed by confocal microscopy (I). Scale bars, 10 μm.

The molecular weights of the catalytic domains of Rgp and Kgp in bacterial culture media are ~44 and 51 kDa, respectively [13,27]. Because the molecular weight of gingipains is comparable to that of 40 kDa dextran, we postulated that gingipains would penetrate gingival epithelial tissues. To test this idea, we employed a two-layered cell culture model to confirm penetration of gingipains from the upper to the lower space (Fig 8A). One hour after infection, the level of JAM1 in the cells of the lower layer was decreased by *P*. *gingivalis* WT to a greater extent than by *P*. *gingivalis* Δ*kgp* Δ*rgpA* Δ*rgpB* (Fig 8B). To exclude the possibility that JAM1 expression had been down-regulated in the lower layer, we used IHGE cells expressing Myc-mCherry–tagged HA-inserted JAM1 in the lower space of the two-layered culture system (Fig 8C). One hour after infection, the level of HA-labeled JAM1 in IHGE cells in the lower layer was decreased by infection with *P*. *gingivalis* WT relative to the mutant, whereas the mCherry signal was not decreased by infection with either strain (Fig 8D), indicating that JAM1 proteins in the lower cells were degraded by *P*. *gingivalis* gingipains, but gene expression was not downregulated. To exclude the possibility that JAM1 in the lower space was degraded by factor(s) other than gingipains, we next examined IHGE cells stably expressing HA-JAM1 Δ(1–133) or Δ(1–133) K134H R234H in the lower space of two-layered culture system (Fig 8E). One hour after infection, the level of HA-labeled JAM1 Δ(1–133) in the lower layer was decreased by infection with *P*. *gingivalis*, but the level of JAM1 Δ(1–133) K134H R234H was unaffected (Fig 8F), indicating that the K134 and R234 residues of JAM1 were digested by gingipains derived from the upper space. To test the possibility that secreted gingipains penetrate gingival epithelial tissues, we used multilayered tissues with IHGE cells in the upper space and IHGE cells expressing Myc-mCherry–tagged HA-inserted JAM1 in the lower space (S14A Fig). Two hours after infection, the level of HA-labeled JAM1 in the cells of the lower layer was decreased by administration of bacterial culture supernatant from *P*. *gingivalis* WT, but not by the mutant, whereas the mCherry signal was not decreased by administration of culture supernatant from either strain (S14B Fig), indicating that JAM1 protein in the lower cells was degraded by secreted gingipains, but expression of the *JAM1* gene was not downregulated. These results strongly suggest that *P*. *gingivalis* gingipains are capable of penetrating the gingival epithelial barrier.

On the basis of these findings, we predicted that overexpression of JAM1 that saturates the capacity of *P*. *gingivalis* to degrade JAM1 would block the loss of JAM1 in gingival epithelial tissues. To test this idea, we performed two-layered culture of IHGE cells overexpressing JAM1, and then analyzed the localization of JAM1 in cells of the lower layer following infection with *P*. *gingivalis* (Fig 8G). Fig 8H shows that when IHGE cells overexpressing JAM1 were infected with *P*. *gingivalis* for 1 h, the remaining JAM1 proteins were present at almost the same level as in non-infected IHGE cells, suggesting that overexpression of JAM1 effectively compensated degradation. Indeed, 1 h after infection, the levels of JAM1 in the lower layer were increased when the cells in the upper layer overexpressed JAM1 (Fig 8I). These results also clearly suggest that degradation of JAM1 by gingipains is involved in penetration of the proteases through the gingival epithelial barrier.

**Fig 9:**
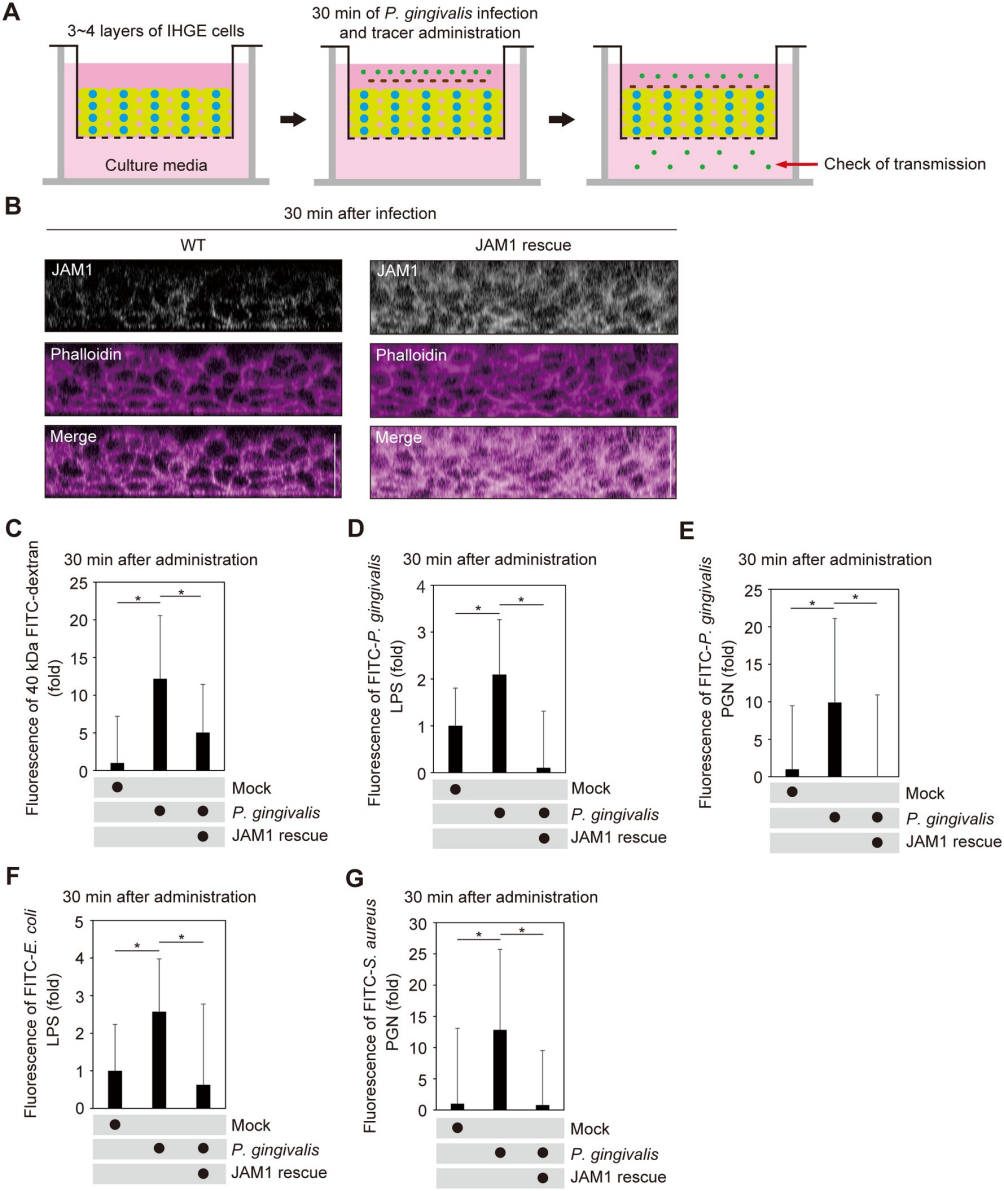
*P*. *gingivalis* degrades JAM1 of gingival epithelium, causing penetration of LPS and PGN. **(A, B)** Schematic illustration of the three-dimensional culture (A) and confocal microscopic cross-sectional images (B) of the three-dimensional culture of IHGE cells. Gingival epithelial tissues (WT or overexpressing JAM1) were infected with *P*. *gingivalis* for 30 min. Tissues were then fixed, stained with anti-JAM1 (white) and Alexa Fluor 568–conjugated phalloidin (magenta), and analyzed by confocal microscopy. Scale bars, 30 μm. **(C**–**G)** Permeability to 40 kDa FITC-dextran (C), FITC–*P*. *gingivalis* LPS (D), FITC–*P*. *gingivalis* PGN (E), FITC–*E*. *coli* LPS (F), and FITC–*S*. *aureus* PGN (G) of gingival epithelial tissues (WT or overexpressing JAM1) infected with *P*. *gingivalis*. Three-dimensional tissues on culture inserts were infected with *P*. *gingivalis* and FITC-labeled tracer in the upper compartment. Following 30 min of incubation, the transmission of tracer from the upper compartment to the lower compartment was analyzed by spectrometry. Results are expressed as fold change relative to uninfected WT cells and are the means ± SD of seven technical replicates. *, p<0.05, one-tailed *t* test (closed testing procedure).

Next, we generated multilayered tissues using IHGE cells overexpressing JAM1 (Fig 9A). Under these conditions, marked amounts of JAM1 remained in tissues infected with *P*. *gingivalis* (Fig 9B). The tissues were then treated with 40 kDa FITC-dextran, FITC–*P*. *gingivalis* LPS, or FITC–*P*. *gingivalis*-PGN and subjected to permeability assays. Thirty minutes after administration, the permeability to 40 kDa FITC-dextran (Fig 9C), FITC–*P*. *gingivalis* LPS (Fig 9D), FITC–*P*. *gingivalis* PGN (Fig 9E), FITC–*E*. *coli* LPS (Fig 9F), and FITC–*S*. *aureus* PGN (Fig 9G) was drastically decreased by overexpression of JAM1 in gingival epithelial tissues. These results suggest that JAM1 degradation by *P*. *gingivalis* plays an important role in allowing the penetration of LPS and PGN in gingival epithelium. To test the possibility that secreted gingipains allow the penetration of LPS and PGN in gingival epithelial tissues, we administered bacterial culture supernatant from *P*. *gingivalis* and FITC-labeled tracer to multilayered tissues containing IHGE cells (S15A Fig). Thirty minutes after administration, the permeability to 40 kDa FITC-dextran (S15B Fig), FITC–*P*. *gingivalis* LPS (S15C Fig), FITC–*P*. *gingivalis* PGN (S15D Fig), FITC–*E*. *coli* LPS (S15E Fig), and FITC–*S*. *aureus* PGN (S15F Fig) was drastically increased following administration of the bacterial culture supernatant of *P*. *gingivalis* WT. These results suggest that gingipains secreted by *P*. *gingivalis* play an important role in allowing the penetration of LPS and PGN in gingival epithelium.

## Discussion

**Fig 10:**
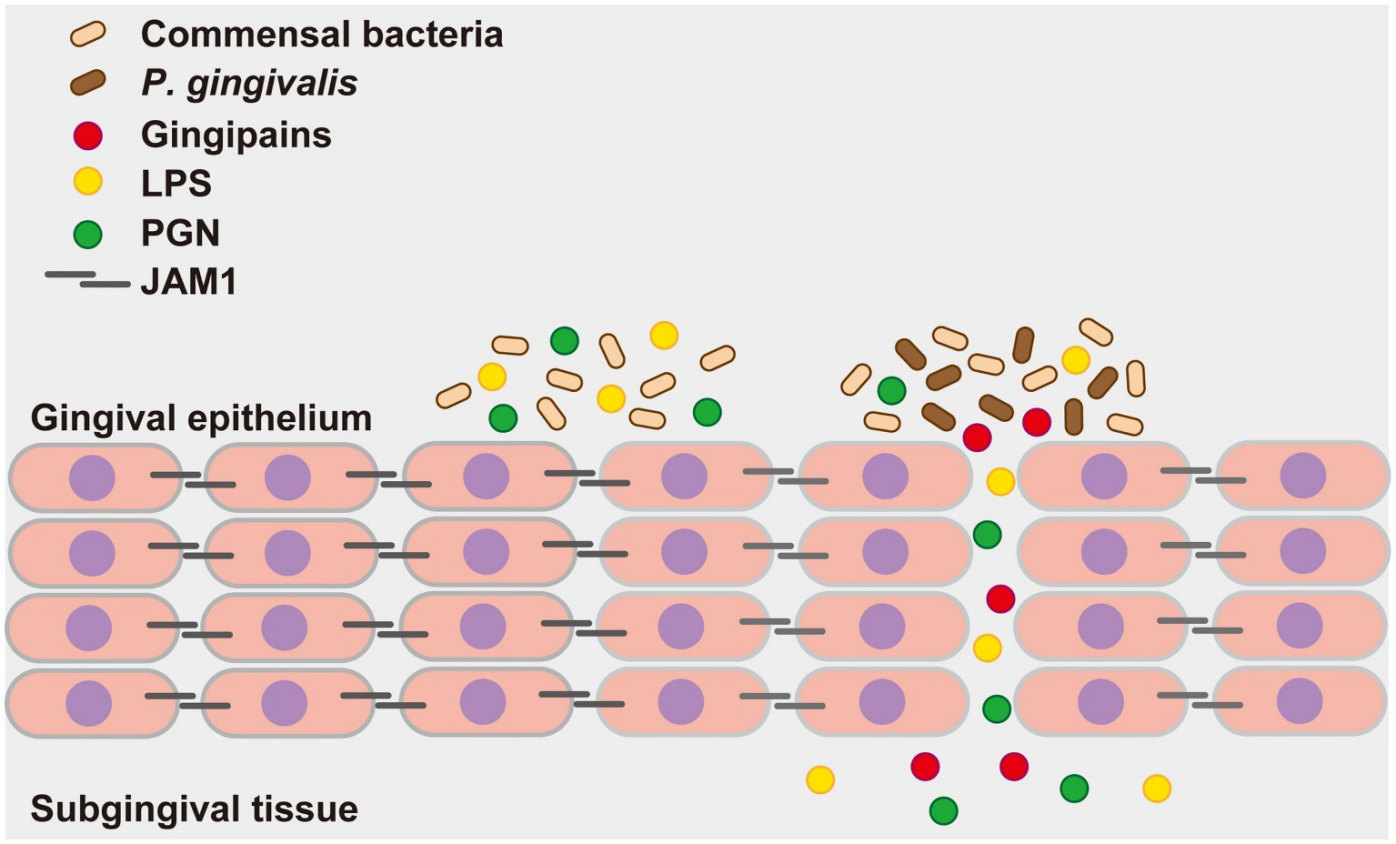
Proposed model of how *P*. *gingivalis* gingipains send bacterial virulence factors through the gingival epithelium. *P*. *gingivalis* gingipains degrade JAM1, which increases the permeability of gingival epithelium to gingipains and other factors. Subsequently, gingipains are transferred to the deeper epithelium to further degrade JAM1, which allows LPS and PGN to penetrate the gingival epithelium and reach subepithelial tissues. Finally, gingipains, LPS, and PGN induce inflammation in gingival tissues.

Our findings clearly suggest a molecular basis for the abrogation of epithelial barrier function by *P*. *gingivalis*. Based on our results, we postulate the following model (Fig 10). By degrading JAM1, *P*. *gingivalis* induces penetration of LPS, PGN, and gingipains derived from itself and other bacteria through the epithelium of the gingival sulcus. The subsequent protein degradation further increases and expands tissue permeability, causing continuous translocation of gingipains in deeper epithelium, leading to periodontal destruction. This cycle could promote an overall increase in the permeability of gingival epithelium, enabling penetration of LPS and PGN into deeper periodontal tissues. This is the first report to reveal the molecular basis of PAMP penetration through human stratified squamous epithelium mediated by a bacterial enzyme. Via this, *P*. *gingivalis* is likely able to destroy the epithelial barrier, thereby remotely causing alveolar bone loss.

In this study, we developed a 3D-tissue model using the cell-accumulation technique by a single cell coating with fibronectin/gelatin nanofilms [22]. This technique has various advantages: healthy, multi-layered human tissues such as stratified squamous epithelium can be developed, and the number of layers is controllable. Manipulation of artificial and acquired genes, including overexpression and knockdown, is also possible. Considering the difficulties of direct observation of transmitted fluorescent tracer in human tissues before disease onset, the epithelium is useful for defining the cause-and-effect relationships of risk factors from the standpoints of quantitative capability, number of samples, repeatability, and research ethics.

We also generated HA-inserted JAM1 for use as a probe in gingival epithelial cells. This probe has several advantages: the tagged protein enables us to monitor degradation by gingipains semi-quantitatively, even when the availability of antibodies is limited; structural analyses of deletion or point mutations are possible; and even when the target protein is post-transcriptionally digested, we can perform flux analysis of degradation by gingipains. Using an HA-tagged protein, we were able to detect the responsible amino acid residues to bacterial degradation.

The full extent of epithelial barrier function in the sulcular epithelium remains to be fully defined. Our immunoblot analyses revealed that JAM1 expression in gingival epithelial cells was clearly suppressed by two sets of specifically designed shJAM1 sequences (Fig 6B). Additionally, we showed that gingival epithelium is selectively impermeable to 40 kDa dextran, LPS, and PGN; this mechanism was also regulated by JAM1 (Fig 7C–7G). Collectively, these results provide evidence that JAM1 plays important roles in subgingival epithelial barrier.

*P*. *gingivalis* has been reported to degrade OCLN and CDH1 in MDCK cellular lysates [10]. By contrast, our findings showed that *P*. *gingivalis* specifically degraded only JAM1 (Fig 1). We assume that this discrepancy is due to our experimental conditions, i.e., infection to living IHGE cells, enabling us to specifically monitor the effects of gingipains on the extracellular domains of host junctional proteins. Our infection methods also exclude artifactual degradation of the intracellular and transmembrane domains of JAM1. Because the immature form of JAM1 in endoplasmic reticulum was not degraded by gingipains (Figs 3C, 3D and 4B), our experiments take into account the spatial limitations of gingipain transmission in gingival epithelial cells.

Homodimer formation of JAM1 is important for localization of JAM1 at tight junctions and for regulation of epithelial barrier function [28]. That study also reported that the JAM1 homodimer interface mutant lacking the N-terminal IG-LIKE domain fails to concentrate at junctions. Because *P*. *gingivalis* degrades JAM1 at the K134 and R234 residues around the C-terminal IG-LIKE domain (Fig 5), the N-terminal IG-LIKE domain is completely separated. Therefore, it seems reasonable that gingipains target K134 and R234 to induce penetration of LPS, PGN, and gingipains.

Another important finding of this study was the observation that gingipains can penetrate the epithelial barrier through degradation of JAM1 (Fig 8). Our results suggest that multiple virulence factors of *P*. *gingivalis*, such as LPS and PGN, can obtain selective permeability through the epithelial barrier due to the actions of gingipains. Thus, these virulence factors can penetrate epithelial barriers and invade deeper connective tissues. In particular gingipains, as predominant virulence factors of *P*. *gingivalis*, can easily invade tissues. The causal relationship between periodontal diseases and systemic diseases is becoming clear. Very recently, gingipains were detected in the brain of Alzheimer’s patients, and the levels were correlated with brain neurodegeneration [29]. That study also reported that gingipains have detrimental effects on tau, a protein required for normal neuronal function, in mice orally infected with *P*. *gingivalis*. The ability of gingipains to invade tissues may thus contribute to Alzheimer’s disease.

This study has revealed the first example of a bacterial protease that can disrupt barrier function of stratified squamous epithelium through degradation of host junctional proteins, leading to penetration of LPS and PGN into subepithelial tissues. Given that multiple PAMPs are expressed by *P*. *gingivalis* [30], JAM1 degradation may enable penetration of the other bacterial product(s) into subepithelial tissues and vessels.

## Materials and methods

### Bacteria and cell culture

*P*. *gingivalis* ATCC 33277 (purchased from the American Type Culture Collection), TDC60 (kindly provided by Kazuyuki Ishihara, Tokyo Dental College), and KDP136 (Δ*kgp* Δ*rgpA* Δ*rgpB*, kindly provided by Koji Nakayama, Nagasaki University) [31] were maintained anaerobically on blood agar plates (BD) and grown in trypticase soy broth (BD), supplemented with hemin (5 μg mL^-1^; Wako), and menadione (1 μg mL^-1^; Sigma-Aldrich). *S*. *gordonii* ATCC 35105 and *F*. *nucleatum* subsp. *nucleatum* ATCC 25586 were cultured as describe previously [32]; *S*. *gordonii* was grown aerobically at 37°C in Todd–Hewitt broth, and *F*. *nucleatum* was grown anaerobically at 37°C in trypticase soy broth (BD) with defibrinated sheep blood. IHGE cells (epi 4, kindly provided by Shinya Murakami, Osaka University) were maintained in Humedia KG-2 (Kurabo), as described previously [33]. For the preparation of the bacterial culture supernatant of *P*. *gingivalis*, bacterial culture at stationary phase was centrifuged at 3,300 *g* for 3 min, and the supernatant was collected and added to the culture media of IHGE cells at a ratio of 1:50.

### Cell accumulation technique

Three-dimensional culture of IHGE cells was performed as described previously [22] with some modification. Briefly, IHGE cells collected by centrifugation after trypsinization were alternatively incubated for 1 min with 0.2 mg mL^-1^ fibronectin (Sigma-Aldrich) and 0.1% (w/v) gelatin (Nacalai Tesque) dissolved in phosphate-buffered saline (PBS). After each procedure, cells were washed with PBS (pH = 7.4) by centrifugation at 180 *g* for 2 min to remove unabsorbed polymers. After nine steps of immersion, fibronectin/gelatin nano-films were coated onto single-cell surfaces. A total of 2 × 10^6^ cells coated with fibronectin/gelatin nano-films were seeded on coverslips coated with 0.2 mg mL^-1^ fibronectin dissolved in PBS in 24-well plates (Iwaki). After 36 h of incubation, tissues were subjected to experiments, fixed, and analyzed on a confocal microscope (TCS SP8; Leica Microsystems). For infection of gingival epithelial tissues, the density of *P*. *gingivalis* cells in culture media was adjusted to OD (600 nm) of 0.12.

### Antibodies and reagents

Mouse monoclonal anti–c-Myc (M4439), mouse monoclonal anti-HA (H3663), mouse-monoclonal anti-JAM1 (SAB4200468), and mouse monoclonal β-Actin (A2228) antibodies were from Sigma-Aldrich; rabbit polyclonal anti-DSC2 (60239-1-Ig) and rabbit polyclonal anti-NECTIN1 (24713-1-AP) antibodies were from Proteintech; rabbit monoclonal anti-HA antibody (3724) and rabbit polyclonal anti-CDH1 (14472) were from Cell Signaling Technology; rabbit polyclonal anti-TOMM20 (ab78547) was from Abcam. Alexa Fluor 488–conjugated secondary (goat anti–mouse IgG, A-11001), Alexa Fluor 568–conjugated secondary (goat anti–mouse IgG, A-11004), and Alexa Fluor 635–conjugated secondary (goat anti–rabbit IgG, A-31576) antibodies (Invitrogen) were used for fluorescence microscopy. Goat anti-mouse (7076) and anti-rabbit (7074) antibodies conjugated to horseradish peroxidase (Cell Signaling Technology) were used for immunoblotting. 4’,6-diamidino-2-phenylindole (DAPI) (D1306, Invitrogen), Alexa Fluor 488–conjugated phalloidin (A12379, Invitrogen), and Alexa Fluor 633–conjugated phalloidin (A22284, Invitrogen) were used to stain IHGE cells.

### Plasmids

The pCMV-Myc-mCherry or pCMV-HA-EGFP plasmid was prepared as described previously [34]. The plasmid encoding Myc-mCherry–tagged HA-inserted JAM1 was constructed by cloning PCR products amplified from IHGE cells into pCMV-Myc-mCherry, using exogenously added *Eco*RI and *Kpn*I sites. To produce HA-inserted JAM1, DNA encoding the HA-tag was inserted into *JAM1* by fusion PCR. Plasmids encoding JAM1 mutants in Fig 5 were constructed by PCR products amplified from WT or HA-inserted JAM into either pCMV-vector (Clontech) or pCMV-HA (Clontech), respectively. Plasmids encoding N-terminal deletion mutants at the signal peptide of JAM1 in S7 Fig were constructed by cloning PCR products amplified from HA-inserted JAM1 into pCMV-Myc-mCherry. Plasmids encoding Myc-mCherry–tagged CLDN1, CLDN4, and OCLN were constructed by cloning PCR products amplified from cells into pCMV-Myc-mCherry, using exogenously added *Eco*RI and *Kpn*I sites. Plasmid encoding HA-tagged TJP1 was constructed by cloning PCR products amplified from cells into pCMV-HA (Clontech), using exogenously added *Eco*RI and *Kpn*I sites. Plasmids encoding EGFP-SEC61β and EGFP-TOMM20 were produced as described previously [34]. PCR amplification was performed with KOD Plus Neo (Kurabo). PCR products were ligated into plasmids with T4 DNA ligase (New England Biolabs). Transfection of IHGE cells was performed using FuGENE 6 Transfection Reagent (Promega).

### Immunoblotting

IHGE cells were lysed, and the lysates were cleared by centrifugation. Proteins were separated by SDS-PAGE and transferred to nitrocellulose membranes (0.2 μm, Bio-Rad). Membranes were blocked for 1 h at room temperature with PBST (PBS and 0.1% (v/v) Tween 20) containing 3% (w/v) skim milk, and then incubated for 1 h at room temperature with primary antibodies diluted in PBST. Next, the membranes were washed three times with PBST and incubated for 1 h at room temperature with 1:5000 dilutions of HRP-conjugated secondary antibodies in PBST. Immunoreactive bands were detected using the Pierce ECL Western Blotting Substrate (Thermo Scientific) and ChemiDoc XRS (Bio-Rad). Images were acquired using the Quantity One software (Bio-Rad).

### Immunocytochemistry

IHGE cells were fixed with 4% (v/v) paraformaldehyde in PBS (Wako) overnight at room temperature, permeabilized with 0.1% (v/v) Triton X-100 (Wako) in PBS for 5 min at room temperature, and blocked with 0.1% (w/v) gelatin (Wako) in PBS for 20 min at room temperature. Samples were incubated for 1 h at room temperature with the indicated primary antibodies, washed four times in PBS, incubated for 1 h at room temperature with the Alexa Fluor–conjugated secondary antibodies (Invitrogen), and again washed four times in PBS. All antibodies were diluted 1:400 in PBS. Cells were mounted onto glass slides with Vectashield Mounting medium (Vector Laboratories). Images were acquired with a confocal laser microscope (TCS SP8; Leica Microsystems) using a 64× oil-immersion object lens with a numerical aperture of 1.4. Acquired images were analyzed using the Application Suite X software (Leica Microsystems).

### Reverse-transcription PCR and real-time PCR

Total RNA was extracted from IHGE cells using TRIzol (Thermo Fisher Scientific). Complementary DNA was synthesized using the iScript cDNA Synthesis Kit (Bio-Rad). Real-time PCR was performed using a Rotor Gene Q (Qiagen) with KAPA SYBR FAST Universal qPCR Kit (KAPA Biosystems). Primer sequences were as follows: *JAM1* forward, 5’-GTGCCTTCAGCAACTCTTCC-3’; reverse, 5’-ACCAGATGCCAAAAACCAAG-3’. *GAPDH* forward, 5’-CCACCCATGGCAAATTCCATGGCA-3’; reverse, 5’-TCTAGACGGCAGGTCAGGTCCACC-3’. Amplicon level in each sample was normalized against the corresponding level of *GAPDH* mRNA content using the 2^−ΔΔCt^ method.

### Generation of cell line stably expressing JAM1

Plasmids encoding JAM1, HA-tagged JAM1 Δ (1–133), and HA-tagged JAM1 Δ (1–133) K134H R234H were constructed by cloning PCR products amplified from the pCMV plasmid into pMRX-IRES-Puro (kindly provided by Nobumichi Furuta, Osaka University) [35]. Plasmids pMRX-IRES-Puro-JAM1, pMRX-IRES-Puro-HA–tagged JAM1 Δ (1–133), and pMRX-IRES-Puro-HA–tagged JAM1 Δ (1–133) K134H R234H were used for overexpression of the cDNA in IHGE cells. IHGE cells were transfected with the overexpression plasmid using FuGENE 6 (Promega). Seventy-two hours after transfection, cells stably overexpressing the cDNA were selected with puromycin (2 μg mL^-1^; InvivoGen).

### RNA interference

Plasmid encoding shRNA was constructed by ligation of linear DNA (Sigma-Aldrich) into pSIREN-RetroQ (Clontech). Plasmids pSIREN-RetroQ-shJAM1 #110 and #508 were used for generation of the siRNA duplex (target sequences: 5’-AAGTCAGAATTCCTGAGAATAAT-3’ and 5’-GGGATAGTGATGCCTACGAATCC-3’, respectively) in cells. Plasmid pSIREN-RetroQ-shLuc was produced as described previously [35]. IHGE cells were transfected with the shRNA-encoding plasmid using FuGENE 6 (Promega). Seventy-two hours after transfection, cells stably expressing the shRNA were selected with puromycin (2 μg mL^-1^).

### Preparation of FITC-labeled tracer

*P*. *gingivalis* LPS (14F18-MM) was purchased from InvivoGen. *P*. *gingivalis* PGN was prepared as described previously [36]. FITC–*E*. *coli* LPS (L7018) and *S*. *aureus* PGN (77140) were purchased from Sigma-Aldrich. Bacterial LPS and PGN were labeled with FITC using Fluorescein Labeling Kit-NH2 (LK-01, Dojindo). Before the assays, FITC-labeled LPS was incubated with 10 mM citrate (Wako) and 0.05% (v/v) Tween-20 (Calbiochem) for 45 min at 37°C as described previously [37], and FITC-labeled PGN was incubated with 0.5 mg mL^-1^ lysozyme (Nacalai Tesque) for 45 min at 37°C to make a suspension. Lucifer yellow (125–06281) was purchased from Wako, and 3–5 kDa FITC-dextran (FD4) and 40 kDa FITC-dextran (FD40) were purchased from Sigma-Aldrich.

### Epithelial barrier function assay

The *in vitro* epithelial permeability assay to assess barrier function was performed with 12-well cell culture inserts (353180; Corning). When IHGE cells in the upper compartment reached 100% confluence, 20 μL of 0.1 mg mL^-1^ Lucifer yellow, FITC-dextran, FITC-LPS, or FITC-PGN was added to the upper compartment of the insert. After a 30-min incubation, the medium was collected from the lower compartment, and the fluorescence intensity was measured using 1420 ARVO *X* (PerkinElmer). Data were acquired using the WorkOut Plus software (PerkinElmer).

### Statistical analysis

P-values were determined by *t* test or Dunnett’s test in Excel (Microsoft); p<0.05 was considered significant.

## Supporting information

S1 Fig *P*. *gingivalis* gingipains in culture supernatant degrade JAM1 in IHGE cells. Bacterial culture supernatant from *P*. *gingivalis* WT or the Δ*kgp ΔrgpA* Δ*rgpB* mutant was administered to IHGE cells. After 1 h of incubation, the cells were analyzed by immunoblotting with the indicated antibodies.
(TIF)

S2 Fig *P*. *gingivalis* do not degrade Myc-mCherry in IHGE cells. IHGE cells were transiently transfected with the Myc-mCherry plasmid. Following 48 h of incubation, cells were infected with *P*. *gingivalis* ATCC 33277 at an MOI of 100 for 1 h or 3 h. The cells were then analyzed by immunoblotting with the indicated antibodies.
(TIF)

S3 Fig Confocal microscopic images of JAM1 in IHGE cells treated with bacterial culture supernatant of *P*. *gingivalis* WT or the Δ*kgp* Δ*rgpA* Δ*rgpB* mutant. **(A)** IHGE cells were treated with bacterial culture supernatant of *P*. *gingivalis* WT or the Δ*kgp* Δ*rgpA* Δ*rgpB* mutant for 1.5 h. The cells were then fixed, stained with DAPI (cyan) and anti-JAM1 (yellow), and analyzed by confocal microscopy. Scale bars, 10 μm. **(B, C)** Schematic illustration (B) and confocal microscopic cross-sectional images (C) of the 3D tissue model of IHGE cells. Gingival epithelial tissues on coverslips were treated with the bacterial culture supernatant of *P*. *gingivalis* WT or the Δ*kgp* Δ*rgpA* Δ*rgpB* mutant for 2 h. The tissues were then fixed, stained with anti-JAM1 (white) and Alexa Fluor 568–conjugated phalloidin (magenta), and analyzed by confocal microscopy. Scale bars, 30 μm.
(TIF)

S4 Fig Confocal microscopic images of artificial gingival epithelial tissue. Epithelial tissues of IHGE cells were fixed, stained with DAPI (cyan) and Alexa Fluor 568–conjugated phalloidin (magenta), and analyzed by confocal microscopy. Scale bar, 30 μm.
(TIF)

S5 Fig Effects of *JAM1* mRNA expression in artificial gingival epithelial tissue. **(A, B)** Schematic illustration (A) and relative *JAM1* mRNA expression (B) in 2D- or 3D-tissue models with IHGE cells. Results are expressed as fold change relative to 2D culture and are the means (cyan bars) of six technical replicates. Significance of differences was evaluated by the two-tailed *t* test.
(TIF)

S6 Fig Confocal microscopic images of an IHGE cell expressing Myc-mCherry–tagged HA-inserted JAM1. IHGE cells were transiently transfected with plasmid encoding Myc-mCherry–tagged HA-inserted JAM1. Following 48 h of incubation, the cells were fixed and stained with anti-JAM1 (green) and anti-HA (cyan), and then analyzed by immunofluorescence microscopy. Scale bar, 5 μm.
(TIF)

S7 Fig Myc-mCherry tag at the N-terminus of JAM1 does not inhibit processing of the signal peptide. **(A)** N-terminal amino acid sequence of JAM1. Magenta font indicates the predicted signal peptide sequence. **(B)** IHGE cells were transiently transfected with a plasmid encoding Myc-mCherry–tagged HA-inserted JAM1 WT or the indicated N-terminal deletion mutant. Following 48 h of incubation, the cells were analyzed by immunoblotting using the indicated antibodies.
(TIF)

S8 Fig HA-inserted JAM1 is secreted to the surface of IHGE cells. **(A, B)** IHGE cells were transiently transfected with plasmid encoding HA-EGFP (A) or Myc-mCherry–tagged HA-inserted JAM1 (B). Following 48 h of incubation, the cells were fixed and stained with anti-HA (cyan), with or without permeabilization, and then analyzed by immunofluorescence microscopy. Scale bars, 5 μm.
(TIF)

S9 Fig Confocal microscopic images of IHGE cells expressing Myc-mCherry–tagged HA-inserted JAM1 and EGFP-SEC61β. **(A–D)** Related to Fig 3C and 3D. Intensities (as determined by the Leica LAS X software) of the fluorescence signals of EGFP-SEC61β (green) and either mCherry or anti-HA (magenta) on the *x*–*y* lines indicated in (A, C) are shown. **(E, F)** IHGE cells were transiently transfected with a plasmid encoding Myc-mCherry–tagged HA-inserted JAM1 (magenta) and EGFP-SEC61β (green). Following 48 h of incubation, the cells were fixed and stained with anti-HA in (F), and then analyzed by immunofluorescence microscopy. Scale bars, 5 μm.
(TIF)

S10 Fig Confocal microscopic images of IHGE cells expressing Myc-mCherry–tagged HA-inserted JAM1 and stained with anti-TOMM20. **(A, B)** IHGE cells were transiently transfected with a plasmid encoding Myc-mCherry–tagged HA-inserted JAM1 (magenta) or EGFP-TOMM20 (green). Following 48 h of incubation, the cells were fixed and stained with anti-HA (B). The cells were then analyzed by immunofluorescence microscopy. **(C, D)** IHGE cells were transiently transfected with a plasmid encoding Myc-mCherry–tagged HA-inserted JAM1 (magenta). Following 48 h of incubation, the cells were fixed and stained with anti-TOMM20 (green) (C, D) or anti-HA (D). The cells were then analyzed by immunofluorescence microscopy. Scale bars, 5 μm.
(TIF)

S11 Fig Confocal microscopy of IHGE cells expressing Myc-mCherry–tagged HA-inserted JAM1 and stained with phalloidin. **(A, B)** IHGE cells were transiently transfected with a plasmid encoding Myc-mCherry–tagged HA-inserted JAM1 (magenta in A, green in B). Following 48 h of incubation, the cells were fixed, stained with Alexa Fluor 488–conjugated phalloidin (green) (A) or Alexa Fluor 633–conjugated phalloidin (magenta) (B), and then analyzed by immunofluorescence microscopy. Scale bars, 5 μm.
(TIF)

S12 Fig *P*. *gingivalis* gingipains in culture supernatant degrade a mature form of JAM1 in IHGE cells. IHGE cells were transiently transfected with the plasmid of Myc-mCherry–tagged HA-inserted JAM1. Following 48 h of incubation, the bacterial culture supernatant of *P*. *gingivalis* WT or the Δ*kgp* Δ*rgpA* Δ*rgpB* mutant was administered to IHGE cells for 1 h. The cells were then analyzed by immunoblotting with the indicated antibodies.
(TIF)

S13 Fig Effects of administration of 40 kDa FITC-dextran on IHGE cells. **(A)** IHGE cells stably expressing shLuc, shJAM1 #110, or shJAM1 #508 were treated with 40 kDa FITC–dextran (green) for 30 min. The cells were then fixed, stained with Alexa Fluor 568–conjugated phalloidin (magenta), and analyzed by confocal microscopy (without permeabilization). Bars, 10 μm. **(B)** Fluorescence (excitation: 485 nm, emission: 535 nm) in IHGE cells with or without administration of 40 kDa FITC–dextran for 30 min. Results are expressed as fold change relative to cells stably expressing shLuc without 40 kDa FITC-dextran and are the means ± SD of eight technical replicates. There was no statistically significant difference (p>0.05, two-tailed *t* test) between the presence and absence of 40 kDa FITC-dextran in any cell line.
(TIF)

S14 Fig *P*. *gingivalis* gingipains penetrate the gingival epithelial tissues. **(A, B)** Schematic image of the culture insert system (A). A multilayer of IHGE cells was cultured in the upper compartment, and a monolayer of IHGE cells expressing Myc-mCherry–tagged HA-inserted JAM1 was cultured on a coverslip in the lower compartment. Bacterial culture supernatant from *P*. *gingivalis* WT or the Δ*kgp* Δ*rgpA* Δ*rgpB* mutant was administered to the tissues in the upper compartment. Following 2 h of incubation, the cells in the lower compartment were fixed, stained with anti-HA (green), and analyzed by confocal microscopy (B). Scale bars, 10 μm.
(TIF)

S15 Fig *P*. *gingivalis* gingipains cause penetration of LPS and PGN. **(A)** Schematic illustration of 3D culture of IHGE cells. FITC tracer and bacterial culture supernatant from *P*. *gingivalis* WT or the Δ*kgp* Δ*rgpA* Δ*rgpB* mutant were administered to the tissues in the upper compartment. Following 30 min of incubation, the transmission of tracer from the upper compartment to the lower compartment was analyzed by spectrometry. **(B**–**F)** Permeability to 40 kDa FITC–dextran (B), FITC–*P*. *gingivalis* LPS (C), FITC–*P*. *gingivalis* PGN (D), FITC–*E*. *coli* LPS (E), and FITC–*S*. *aureus* PGN (F) of gingival epithelial tissues treated with bacterial culture supernatant from *P*. *gingivalis* WT or the Δ*kgp* Δ*rgpA* Δ*rgpB* mutant. Results are expressed as fold change relative to Mock and are the means ± SD of six technical replicates. *, p<0.05, one-tailed *t* test (closed testing procedure).
(TIF)

S16 Fig Immunoblots performed in this study. (PDF)

## References

[1] Eke PI, Dye BA, Wei I, Thornton-Evans GO, Genco RJ. Prevalence of periodontitis in adults in the United States: 2009 and 2010. J Dent Res. 2009; 91: 914–920. 10.1177/0022034512457373 22935673

[2] Dixon DR, Bainbridge BW, Darveau RP. Modulation of the innate response within the periodontium. Periodontol 2000. 2004; 35:53–74. 15107058 10.1111/j.0906-6713.2004.003556.x

[3] Kawai T, Akira S. Toll-like receptors and their crosstalk with other innate receptors in infection and immunity. Immunity. 2011; 34: 637–650. 10.1016/j.immuni.2011.05.006 21616434

[4] König J, Wells J, Cani PD, García-Ródenas CL, MacDonald T, Mercenier A, et al Human intestinal barrier function in health and disease. Clin Transl Gastroenterol. 2016; 7: e196 10.1038/ctg.2016.54 27763627 PMC5288588

[5] Wolf AJ, Underhill DM. Peptidoglycan recognition by the innate immune system. Nat Rev Immunol. 2018; 18: 243–254. 10.1038/nri.2017.136 29292393

[6] Belibasakis GN, Kast JI, Thurnheer T, Akdis CA, Bostanci N. The expression of gingival epithelial junctions in response to subgingival biofilms. Virulence. 2015; 6: 704–709. 10.1080/21505594.2015.1081731 26305580 PMC4720238

[7] Pussinen PJ, Vilkuna-Rautiainen T, Alfthan G, Palosuo T, Jauhiainen M, Sundvall J, et al Severe periodontitis enhances macrophage activation via increased serum lipopolysaccharide. Arterioscler Thromb Vasc Biol. 2004; 24: 2174–2180. 10.1161/01.ATV.0000145979.82184.9f 15388525

[8] Shaddox LM, Wiedey J, Calderon NL, Magnusson I, Bimstein E, Bidwell JA, et al Local inflammatory markers and systemic endotoxin in aggressive periodontitis. J Dent Res. 2011; 90: 1140–1144. 10.1177/0022034511413928 21730256 PMC3169885

[9] Lamont RJ, Koo H, Hajishengallis G. The oral microbiota: dynamic communities and host interactions. Nat Rev Microl. 2018; 16: 745–759. 10.1038/s41579-018-0089-x PMC6278837 30301974

[10] Katz J, Sambandam V, Wu JH, Michalek SM, Balkovetz DF. Characterization of *Porphyromonas gingivalis*-induced degradation of epithelial cell junctional complexes. Infect Immun. 2000; 68: 1441–1449. 10.1128/iai.68.3.1441-1449.2000 10678958 PMC97299

[11] Potempa J, Pike R, Travis J. The multiple forms of trypsin-like activity present in various strains of *Porphyromonas gingivalis* are due to the presence of either Arg-gingipain or Lys-gingipain. Infect Immun. 1995; 63: 1176–1182. 7890369 10.1128/iai.63.4.1176-1182.1995 PMC173131

[12] Nakayama K, Kadowaki T, Okamoto K, Yamamoto K. Construction and characterization of arginine-specific cysteine proteinase (Arg-gingipain)-deficient mutants of *Porphyromonas gingivalis*. Evidence for significant contribution of Arg-gingipain to virulence. J Biol Chem. 1995; 270: 23619–23626. 10.1074/jbc.270.40.23619 7559528

[13] Kadowaki T, Yoneda M, Okamoto K, Maeda K, Yamamoto K. Purification and characterization of a novel arginine-specific cysteine proteinase (argingipain) involved in the pathogenesis of periodontal disease from the culture supernatant of *Porphyromonas gingivalis*. J Biol Chem. 1994; 269: 21371–21378. 8063764

[14] Baba A, Abe N, Kadowaki T, Nakanishi H, Ohishi M, Asao T, et al Arg-gingipain responsible for the degradation of cell adhesion molecules of human gingival fibroblasts and their death induced by *Porphyromonas gingivalis*. Biol Chem. 2005; 382: 817–824. 10.1515/BC.2001.099 11517936

[15] Katz J, Yang QB, Zhang P, Potempa J, Travis J, Michalek SM, et al Hydrolysis of epithelial junctional proteins by *Porphyromonas gingivalis* gingipains. Infect Immun. 2002; 70: 2512–2518. 10.1128/IAI.70.5.2512-2518.2002 11953390 PMC127922

[16] Tsukita S, Furuse M, Itoh M. Multifunctional strands in tight junctions. Nat Rev Mol Cell Biol. 2001; 2:285–293. 11283726 10.1038/35067088

[17] Martìn-Padura I, Lostaglio S, Schneemann M, Williams L, Romano M, Fruscella P, et al Junctional adhesion molecule, a novel member of the immunoglobulin superfamily that distributes at intercellular junctions and modulates monocyte transmigration. J Cell Biol. 1998; 142: 17–127. 10.1083/jcb.142.1.117 PMC2133024 9660867

[18] Ye P, Yu H, Simonian M, Hunter N. Expression patterns of tight junction components induced by CD24 in an oral epithelial cell-culture model correlated to affected periodontal tissues. J Periodont Res. 2014; 49: 253–259. 10.1111/jre.12102 23713517

[19] Liang TW, DeMarco RA, Mrsny RJ, Gurney A, Gray A, Hooley J, et al Characterization of huJAM1: evidence for involvement in cell-cell contact and tight junction regulation. Am J Physiol Cell Physiol. 2000; 279: C1733–1743. 10.1152/ajpcell.2000.279.6.C1733 11078687

[20] Liu Y, Nusrat A, Schnell FJ, Reaves TA, Walsh S, Pochet M, et al Human junction adhesion molecule regulates tight junction resealing in epithelia. J Cell Sci. 2000; 113: 2363–2374. 10852816 10.1242/jcs.113.13.2363

[21] Mandell KJ, Babbin BA, Nusrat A, Parkos CA. Junctional adhesion molecule 1 regulates epithelial cell morphology through effects on β1 integrins and Rap1 activity. J Biol Chem. 2005; 280: 11665–11674. 10.1074/jbc.M412650200 15677455

[22] Nishiguchi A, Yoshida H, Matsusaki M, Akashi M. Rapid construction of three-dimensional multilayered tissues with endothelial tube network by the cell-accumulation technique. Adv Mater. 2011; 23: 3506–3510. 10.1002/adma.201101787 21728193

[23] Naik UP, Naik MU, Eckfeld K, Martin-DeLeon P, Spychala J. Characterization and chromosomal localization of JAM-1, a platelet receptor for a stimulatory monoclonal antibody. J Cell Sci. 2000; 114: 539–547. 10.1242/jcs.114.3.539 11171323

[24] Watanabe T, Maruyama F, Nozawa T, Aoki A, Okano S, Shibata Y, et al Complete genome sequence of the bacterium *Porphyromonas gingivalis* TDC60, which causes periodontal disease. J Bacteriol. 2011; 193: 4259–4260. 10.1128/JB.05269-11 21705612 PMC3147703

[25] Kolenbrander PE, Andersen RN, Blehert DS, Egland PG, Foster JS, Palmer RJ Jr. Communication among oral bacteria. Microbiol Mol Biol Rev. 2002; 66: 486–505. 10.1128/MMBR.66.3.486-505.2002 12209001 PMC120797

[26] Ribi E, Anacker RL, Brown R, Haskins WT, Malmgren B, Milner KC, et al Reaction of endotoxin and surfactants. J Bacteriol. 1966; 92: 1493–1509. 4288609 10.1128/jb.92.5.1493-1509.1966 PMC276450

[27] Okamoto K, Kadowaki T, Nakayama K, Yamamoto K. Cloning and sequencing of the gene encoding a novel lysine-specific cysteine proteinase (Lys-gingipain) in *Porphyromonas gingivalis*: structural relationship with the arginine-specific cysteine proteinase (Arg-gingipain). J Biochem. 1996; 120: 398–406. 10.1093/oxfordjournals.jbchem.a021426 8889827

[28] Mandell KJ, McCall IC, Parkos CA. Involvement of the junctional adhesion molecule-1 (JAM1) homodimer interface in regulation of epithelial barrier function. J Biol Chem. 2004; 279:16254–16262. 10.1074/jbc.M309483200 14749337

[29] Dominy SS, Lynch C, Ermini F, Benedyk M, Marczyk A, Konradi A, et al *Porphyromonas gingivalis* in Alzheimer’s disease brains: Evidence for disease causation and treatment with small-molecule inhibitors. Sce Adv. 2019; 5:eaau3333. 10.1126/sciadv.aau3333 PMC6357742 30746447

[30] Sochalska M, Potempa J. Manipulation of neutrophils by *Porphyromonas gingivalis* of periodontitis. Front Cell Infect Microbiol. 2017; 23: 197. 10.3389/fcimb.2017.00197 PMC5440471 28589098

[31] Shi Y, Ratnayake DB, Okamoto K, Abe N, Yamamoto K, Nakayama K. Genetic analysis of proteolysis, hemoglobin binding, and hemagglutination of *Porphyromonas gingivalis*. Construction of mutants with a combination of rgpA, rgpB, kgp, and hagA. J Biol Chem. 1999; 274: 17955–17960. 10.1074/jbc.274.25.17955 10364243

[32] Sakanaka A, Kuboniwa M, Takeuchi H, Hashino E, Amano A. Arginine-ornithine antiporter ArcD controls arginine metabolism and interspecies biofilm development of *Streptococcus gordonii*. J Biol Chem. 2015; 290: 21185–21198. 10.1074/jbc.M115.644401 26085091 PMC4571851

[33] Murakami S, Yoshimura N, Koide H, Watanabe J, Takedachi M, Terakura M, et al Activation of adenosine-receptor-enhanced iNOS mRNA expression by gingival epithelial cells. J Dent Res. 2002; 81: 236–240. 10.1177/154405910208100403 12097306

[34] Takeuchi H, Takada A, Kuboniwa M, Amano A. Intracellular periodontal pathogen exploits recycling pathway to exit from infected cells. Cell Microbiol. 2016; 18: 928–948. 10.1111/cmi.12551 26617273

[35] Matsunaga K, Saitoh T, Tabata K, Omori H, Satoh T, Kurotori N, et al Two Beclin 1-binding proteins, Atg14L and Rubicon, reciprocally regulate autophagy at different stages. Nat Cell Biol. 2009; 11: 385–396. 10.1038/ncb1846 19270696

[36] Ishii K, Hamamoto H, Imamura K, Adachi T, Shoji M, Nakayama K, et al *Porphyromonas gingivalis* peptidoglycans induce excessive activation of the innate immune system in silkworm larvae. J Biol Chem. 2010; 285: 33338–33347. 10.1074/jbc.M110.112987 20702417 PMC2963355

[37] Reich J, Lang P, Grallert H, Motschmann H. Masking of endotoxin in surfactant samples: Effects on Limulus-based detection systems. Biologicals. 2016; 44: 417–422. 10.1016/j.biologicals.2016.04.012 27464990

