# Supplementary figures and images for: Porphyromonas gingivalis induces penetration of lipopolysaccharide and peptidoglycan through the gingival epithelium via degradation of junctional adhesion molecule 1
Source: PLoS Pathog. 2019 Nov 7;15(11):e1008124. doi: 10.1371/journal.ppat.1008124 (PMC6932823; doi:10.1371/journal.ppat.1008124)

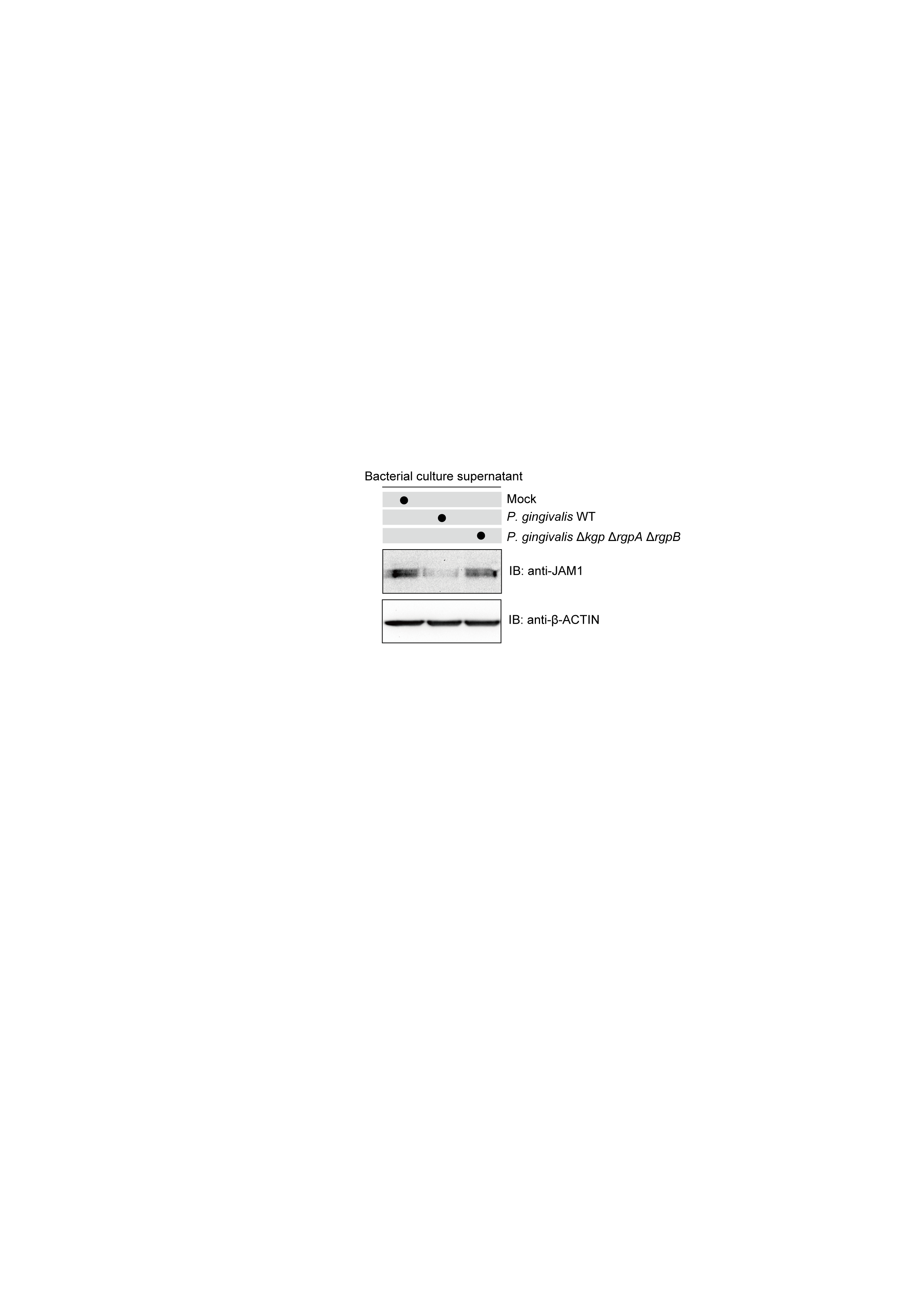

Supplement: S1 Fig — Bacterial culture supernatant from P. gingivalis WT or the Δkgp ΔrgpA ΔrgpB mutant was administered to IHGE cells. After 1 h of incubation, the cells were analyzed by immunoblotting with the indicated antibodies. (TIF) [file ppat.1008124.s001.tif]

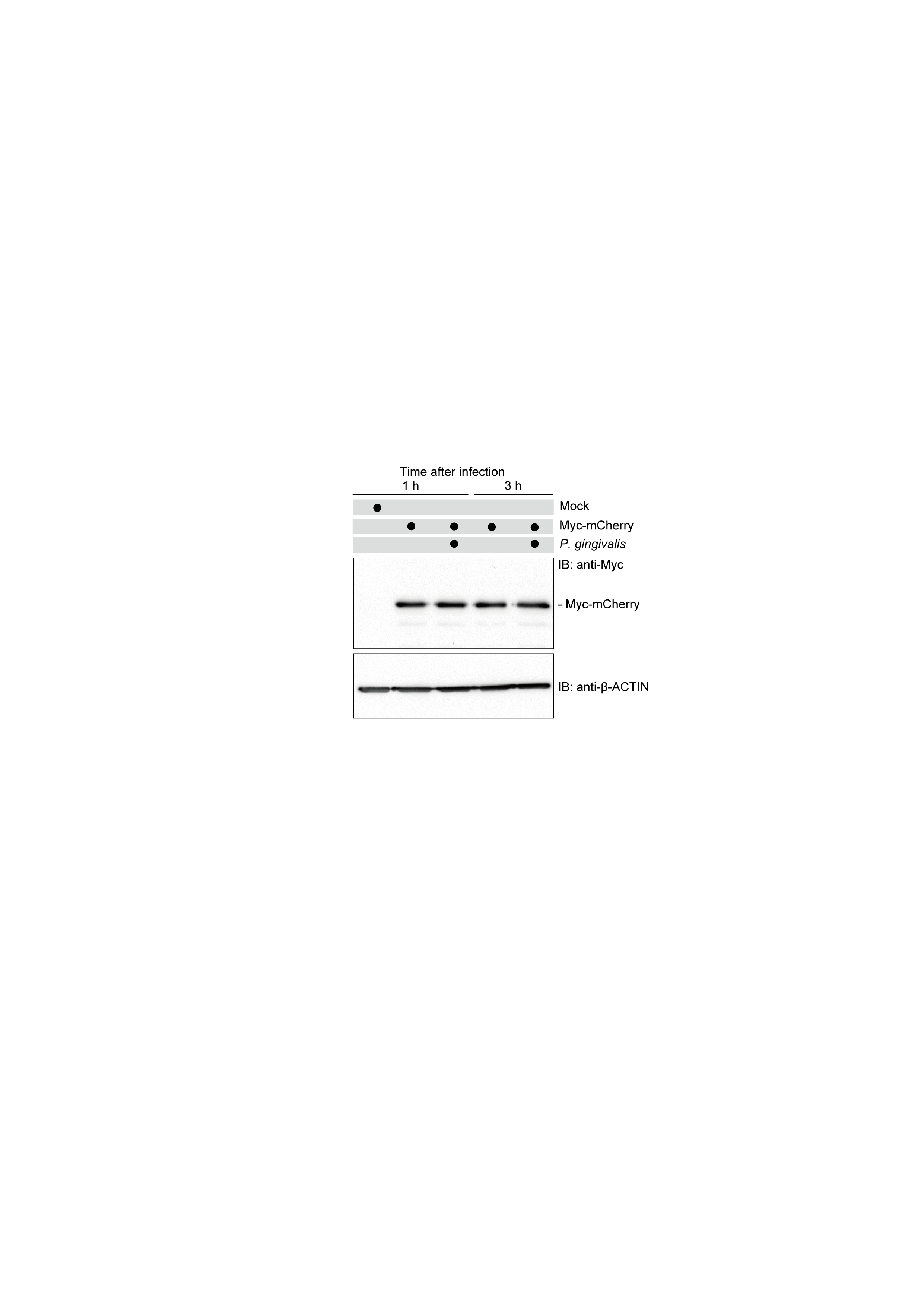

Supplement: S2 Fig — IHGE cells were transiently transfected with the Myc-mCherry plasmid. Following 48 h of incubation, cells were infected with P. gingivalis ATCC 33277 at an MOI of 100 for 1 h or 3 h. The cells were then analyzed by immunoblotting with the indicated antibodies. (TIF) [file ppat.1008124.s002.tif]

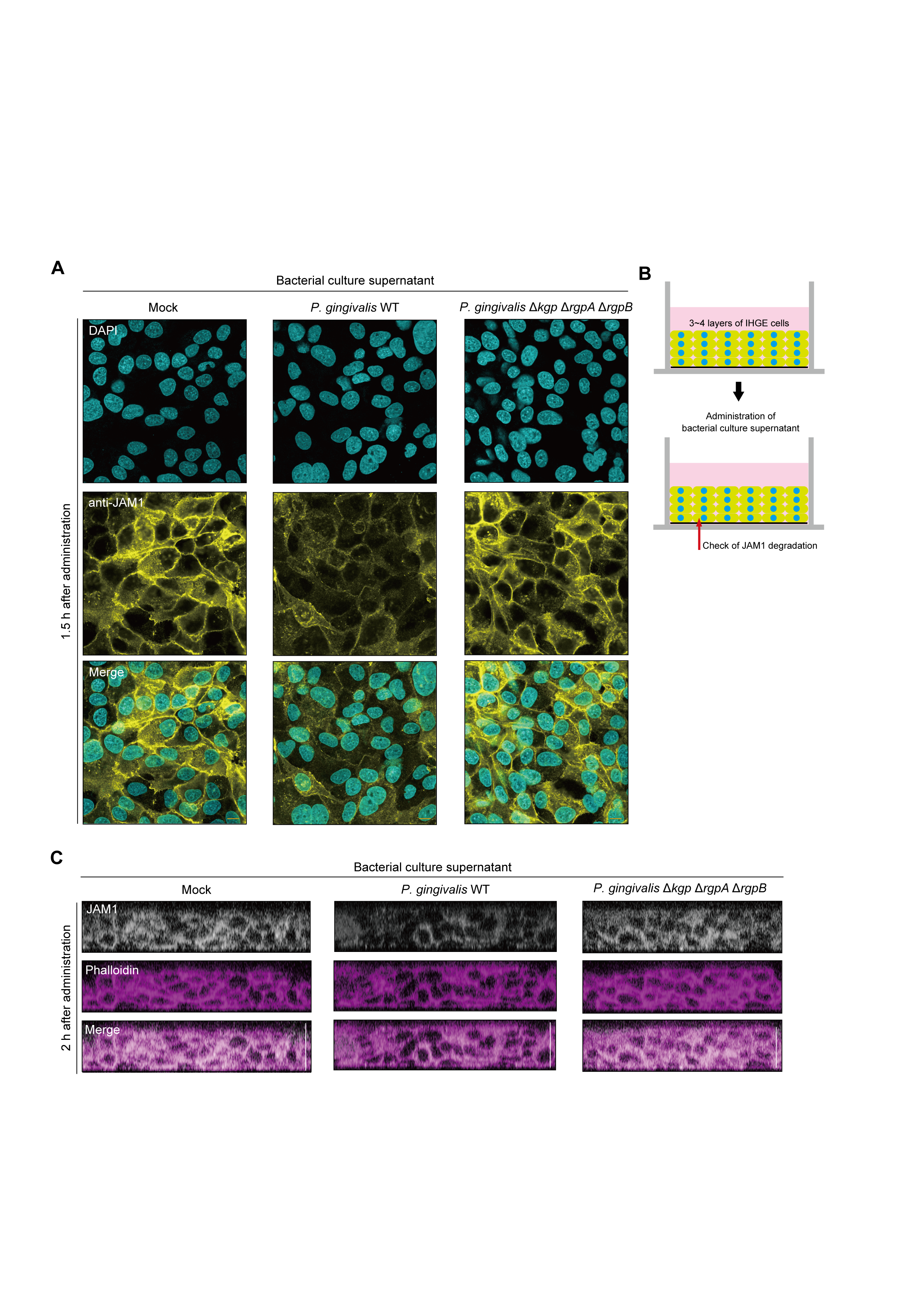

Supplement: S3 Fig — (A) IHGE cells were treated with bacterial culture supernatant of P. gingivalis WT or the Δkgp ΔrgpA ΔrgpB mutant for 1.5 h. The cells were then fixed, stained with DAPI (cyan) and anti-JAM1 (yellow), and analyzed by confocal microscopy. Scale bars, 10 μm. (B, C) Schematic illustration (B) and confocal microscopic cross-sectional images (C) of the 3D tissue model of IHGE cells. Gingival epithelial tissues on coverslips were treated with the bacterial culture supernatant of P. gingivalis WT or the Δkgp ΔrgpA ΔrgpB mutant for 2 h. The tissues were then fixed, stained with anti-JAM1 (white) and Alexa Fluor 568–conjugated phalloidin (magenta), and analyzed by confocal microscopy. Scale bars, 30 μm. (TIF) [file ppat.1008124.s003.tif]

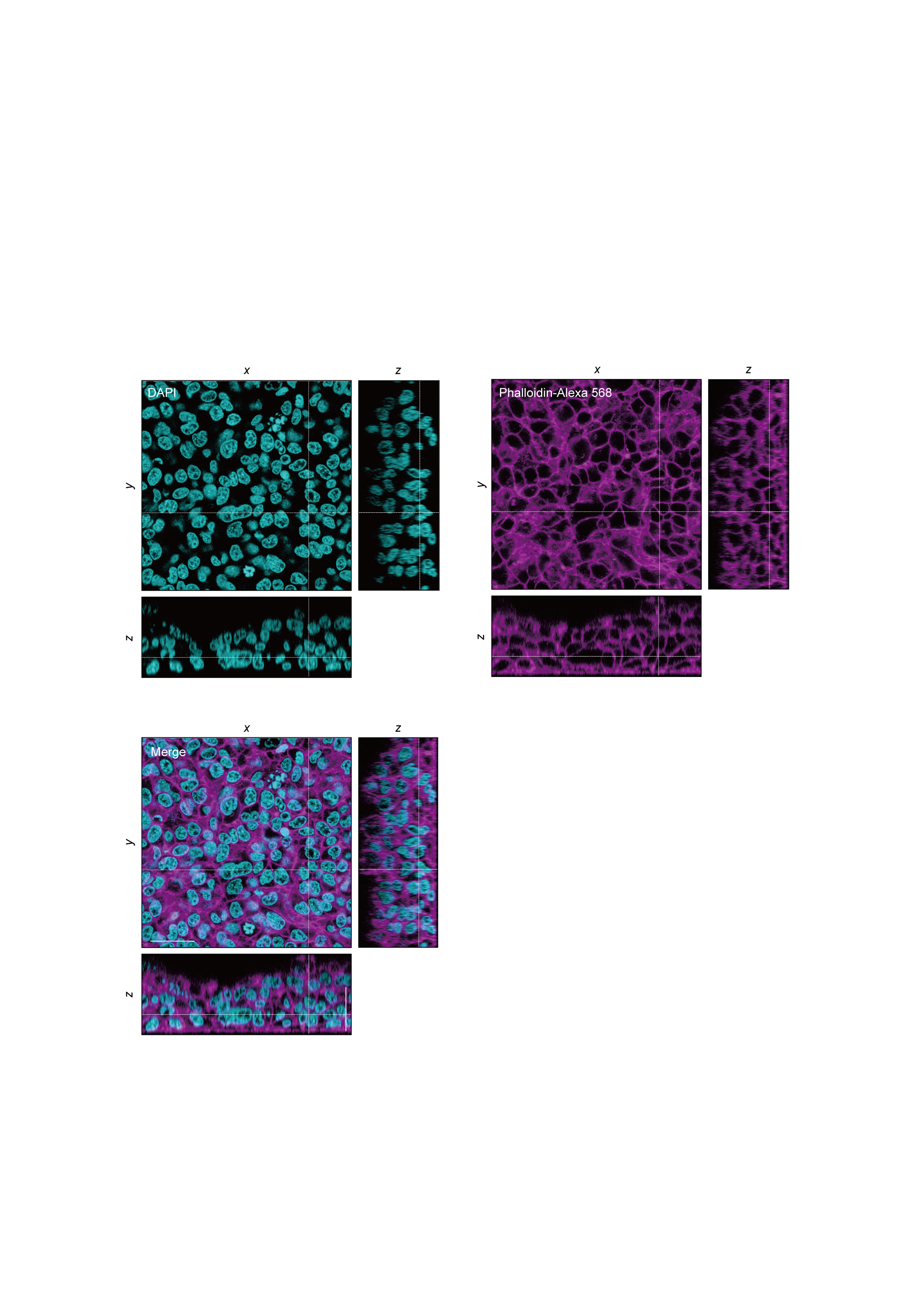

Supplement: S4 Fig — Epithelial tissues of IHGE cells were fixed, stained with DAPI (cyan) and Alexa Fluor 568–conjugated phalloidin (magenta), and analyzed by confocal microscopy. Scale bar, 30 μm. (TIF) [file ppat.1008124.s004.tif]

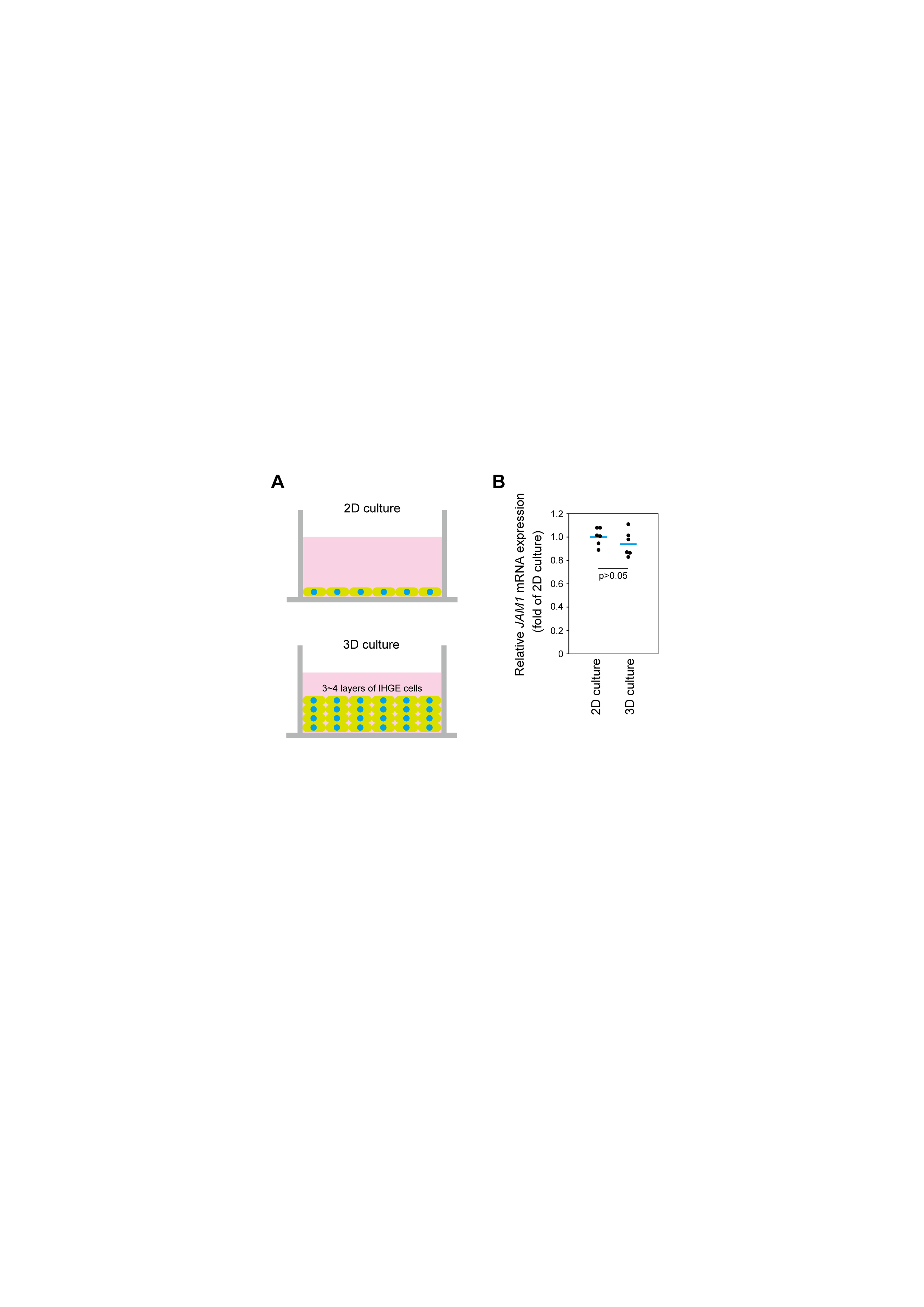

Supplement: S5 Fig — (A, B) Schematic illustration (A) and relative JAM1 mRNA expression (B) in 2D- or 3D-tissue models with IHGE cells. Results are expressed as fold change relative to 2D culture and are the means (cyan bars) of six technical replicates. Significance of differences was evaluated by the two-tailed t test. (TIF) [file ppat.1008124.s005.tif]

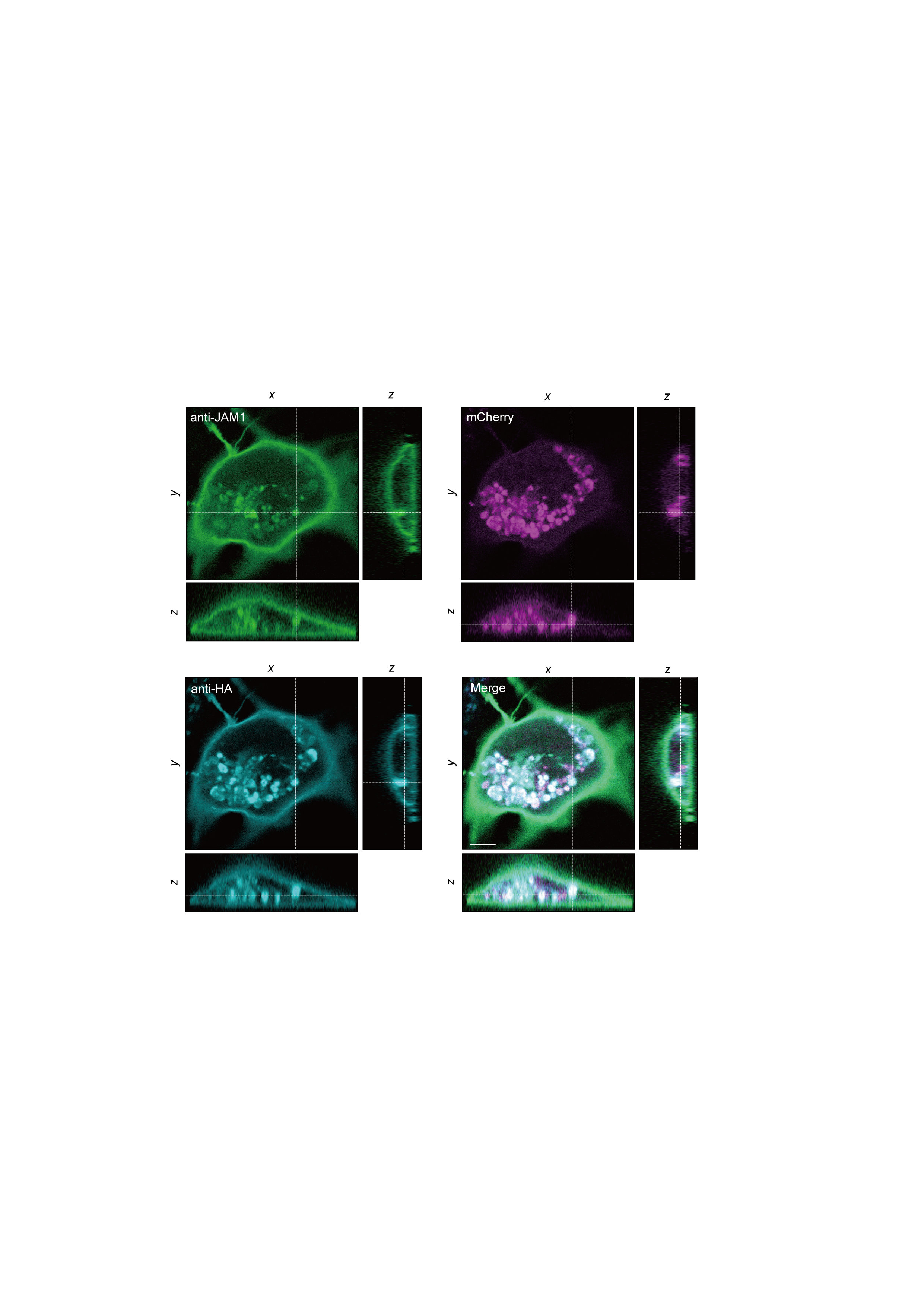

Supplement: S6 Fig — IHGE cells were transiently transfected with plasmid encoding Myc-mCherry–tagged HA-inserted JAM1. Following 48 h of incubation, the cells were fixed and stained with anti-JAM1 (green) and anti-HA (cyan), and then analyzed by immunofluorescence microscopy. Scale bar, 5 μm. (TIF) [file ppat.1008124.s006.tif]

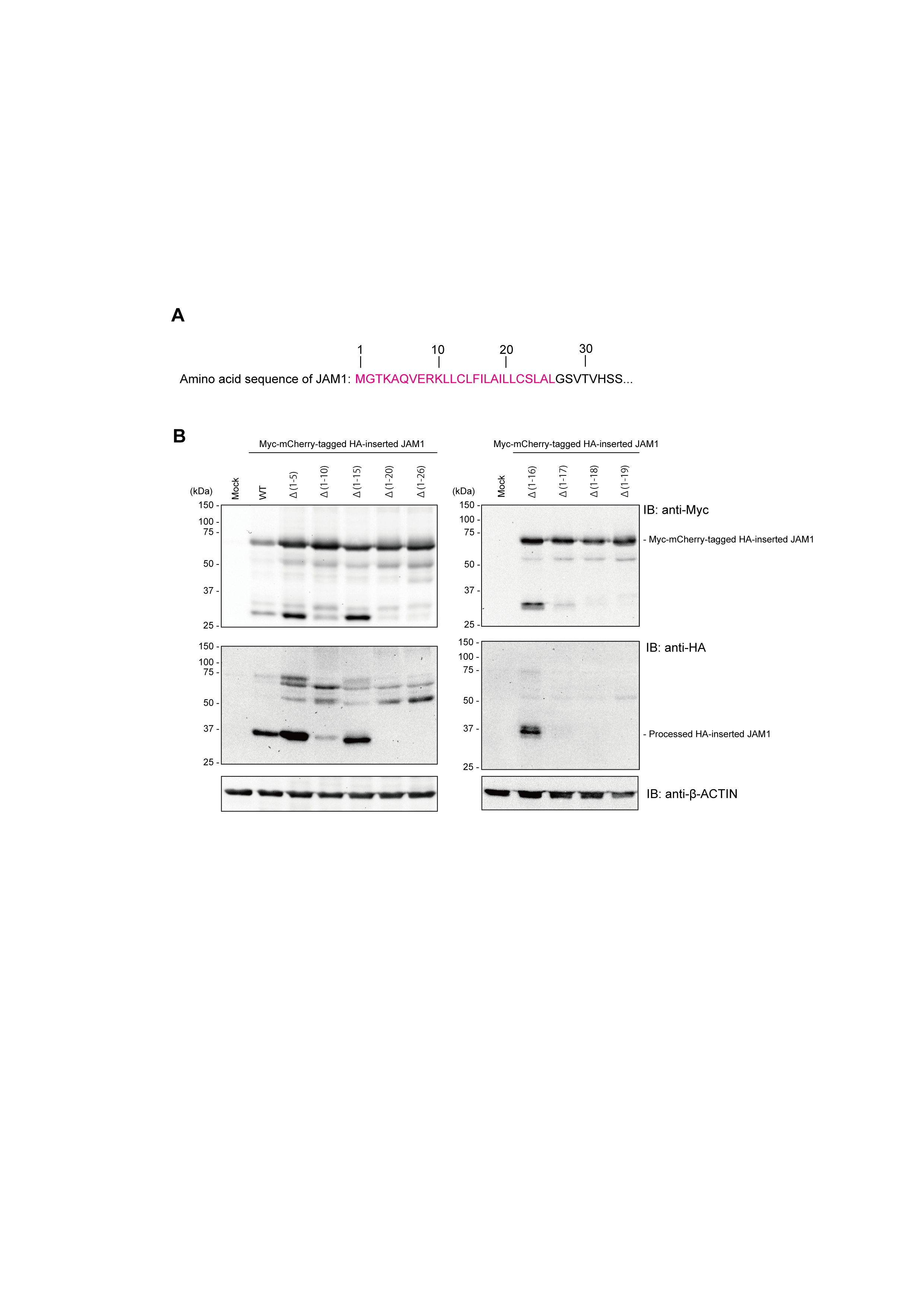

Supplement: S7 Fig — (A) N-terminal amino acid sequence of JAM1. Magenta font indicates the predicted signal peptide sequence. (B) IHGE cells were transiently transfected with a plasmid encoding Myc-mCherry–tagged HA-inserted JAM1 WT or the indicated N-terminal deletion mutant. Following 48 h of incubation, the cells were analyzed by immunoblotting using the indicated antibodies. (TIF) [file ppat.1008124.s007.tif]

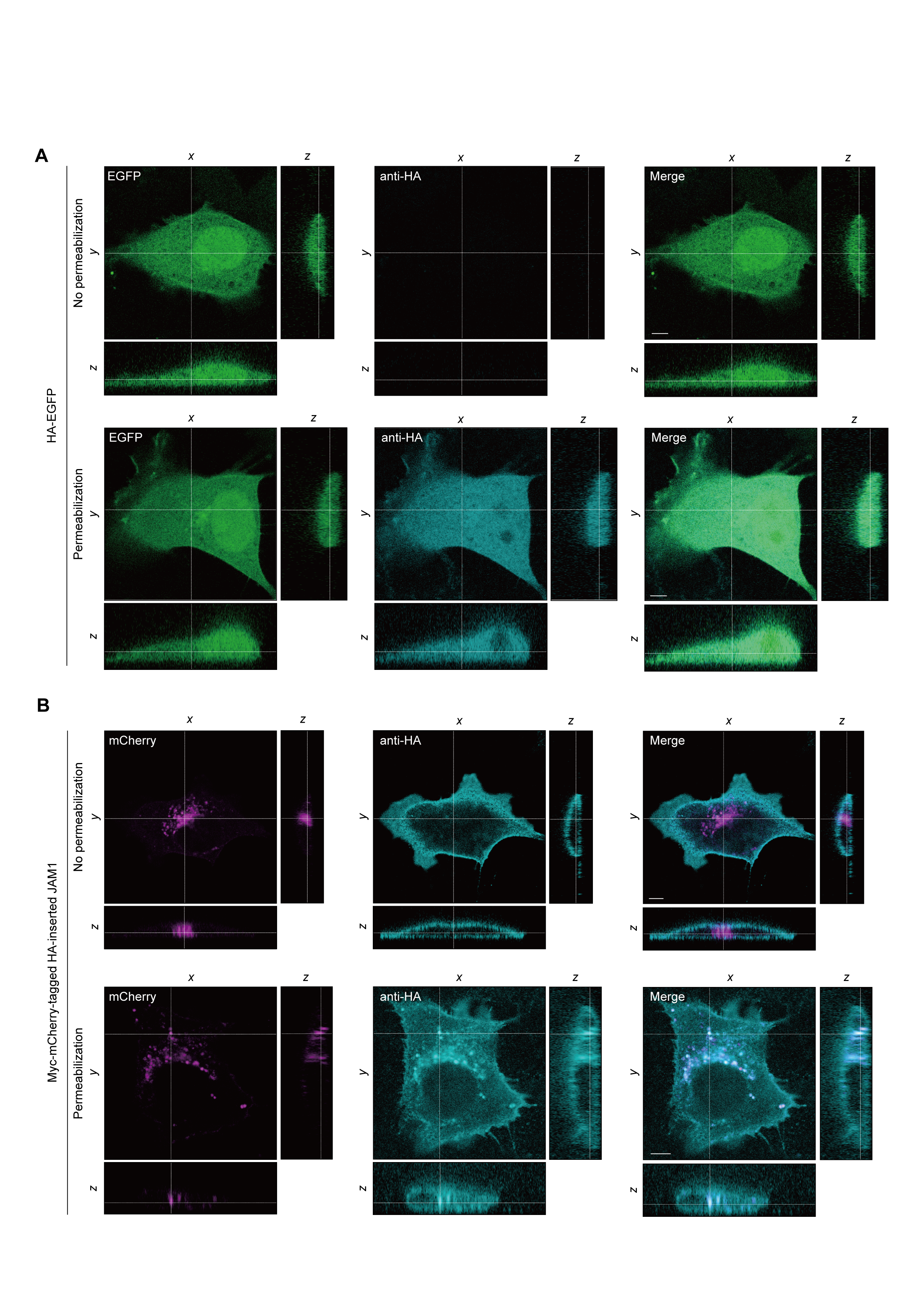

Supplement: S8 Fig — (A, B) IHGE cells were transiently transfected with plasmid encoding HA-EGFP (A) or Myc-mCherry–tagged HA-inserted JAM1 (B). Following 48 h of incubation, the cells were fixed and stained with anti-HA (cyan), with or without permeabilization, and then analyzed by immunofluorescence microscopy. Scale bars, 5 μm. (TIF) [file ppat.1008124.s008.tif]

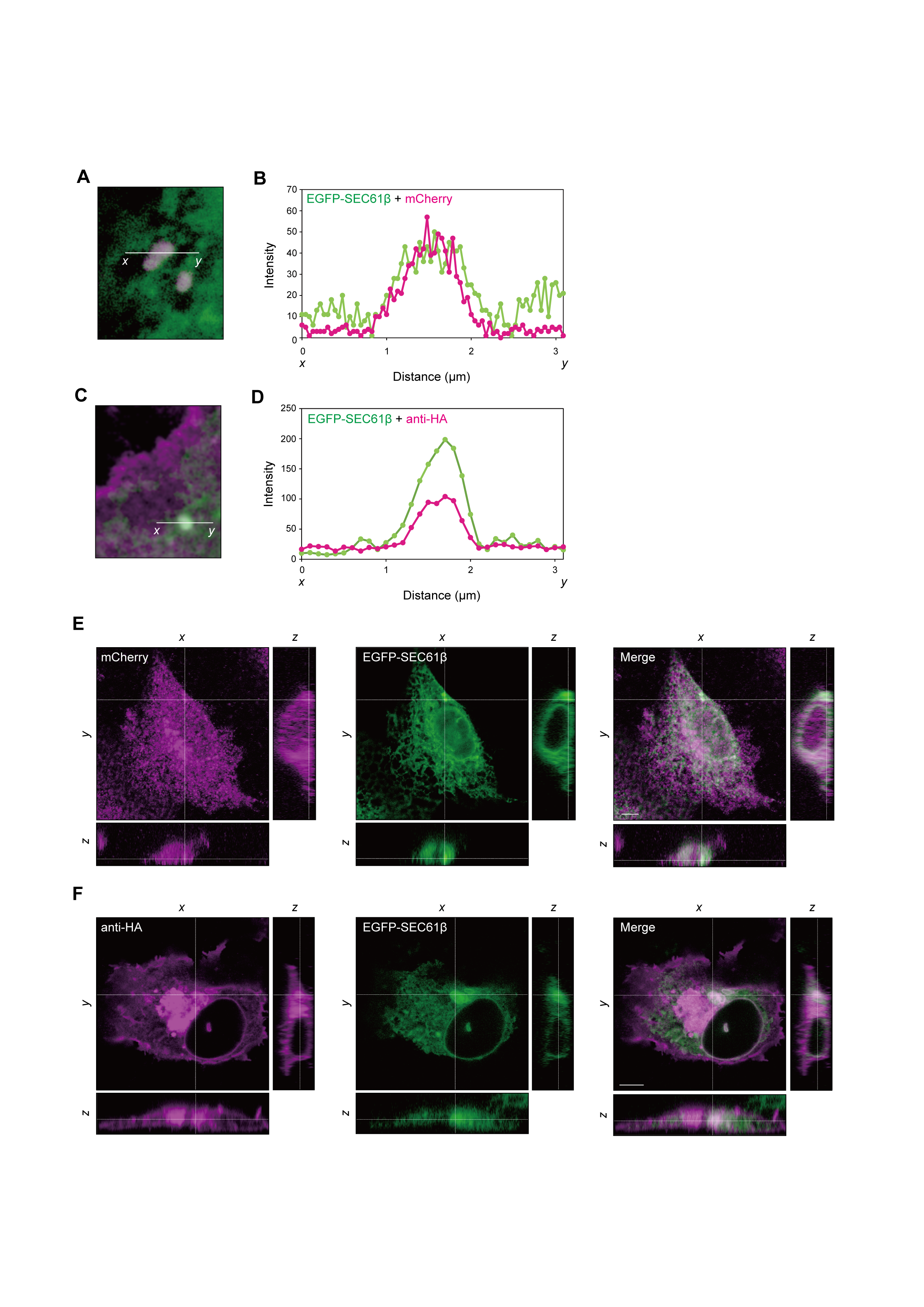

Supplement: S9 Fig — (A–D) Related to Fig 3C and 3D. Intensities (as determined by the Leica LAS X software) of the fluorescence signals of EGFP-SEC61β (green) and either mCherry or anti-HA (magenta) on the x–y lines indicated in (A, C) are shown. (E, F) IHGE cells were transiently transfected with a plasmid encoding Myc-mCherry–tagged HA-inserted JAM1 (magenta) and EGFP-SEC61β (green). Following 48 h of incubation, the cells were fixed and stained with anti-HA in (F), and then analyzed by immunofluorescence microscopy. Scale bars, 5 μm. (TIF) [file ppat.1008124.s009.tif]

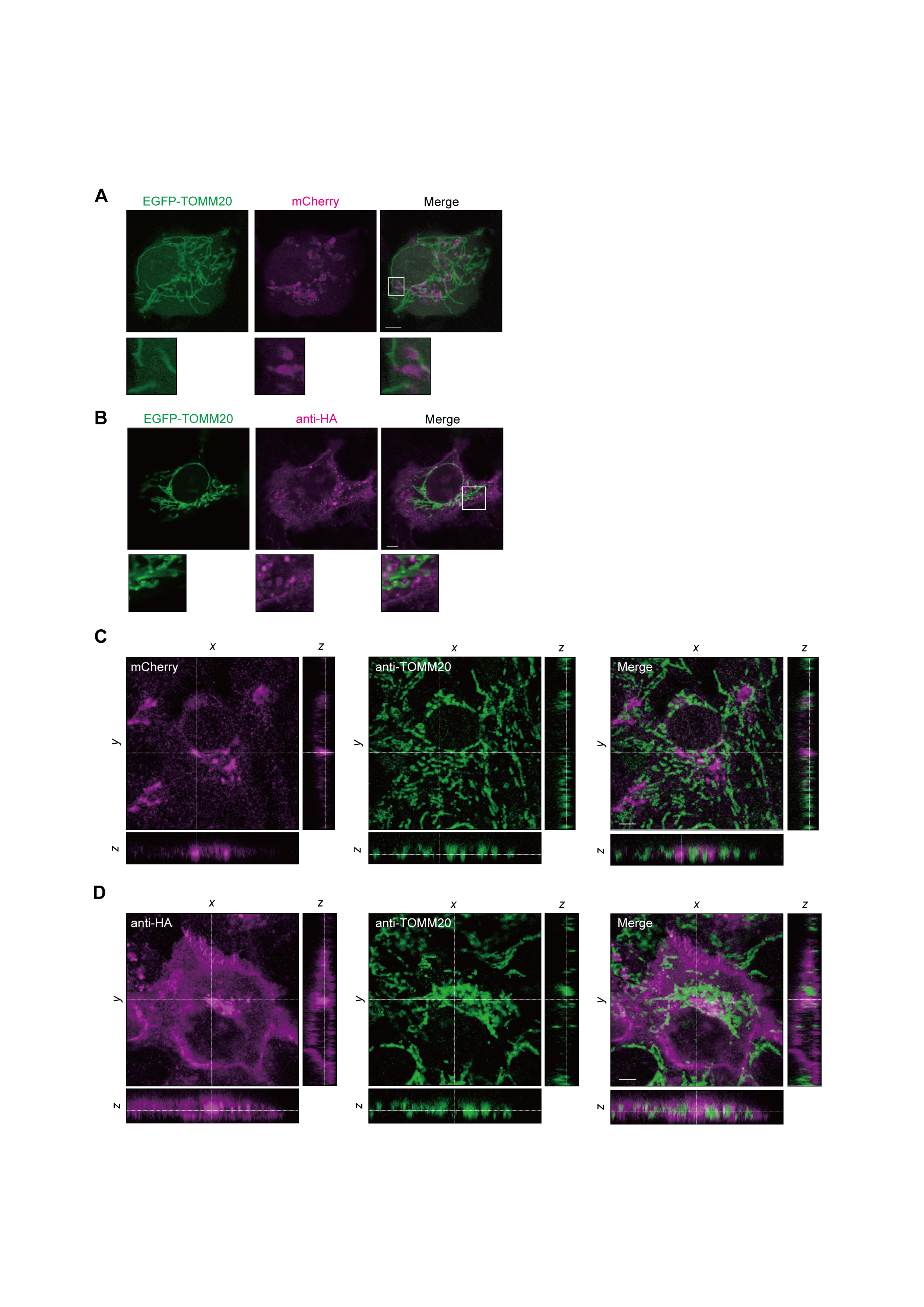

Supplement: S10 Fig — (A, B) IHGE cells were transiently transfected with a plasmid encoding Myc-mCherry–tagged HA-inserted JAM1 (magenta) or EGFP-TOMM20 (green). Following 48 h of incubation, the cells were fixed and stained with anti-HA (B). The cells were then analyzed by immunofluorescence microscopy. (C, D) IHGE cells were transiently transfected with a plasmid encoding Myc-mCherry–tagged HA-inserted JAM1 (magenta). Following 48 h of incubation, the cells were fixed and stained with anti-TOMM20 (green) (C, D) or anti-HA (D). The cells were then analyzed by immunofluorescence microscopy. Scale bars, 5 μm. (TIF) [file ppat.1008124.s010.tif]

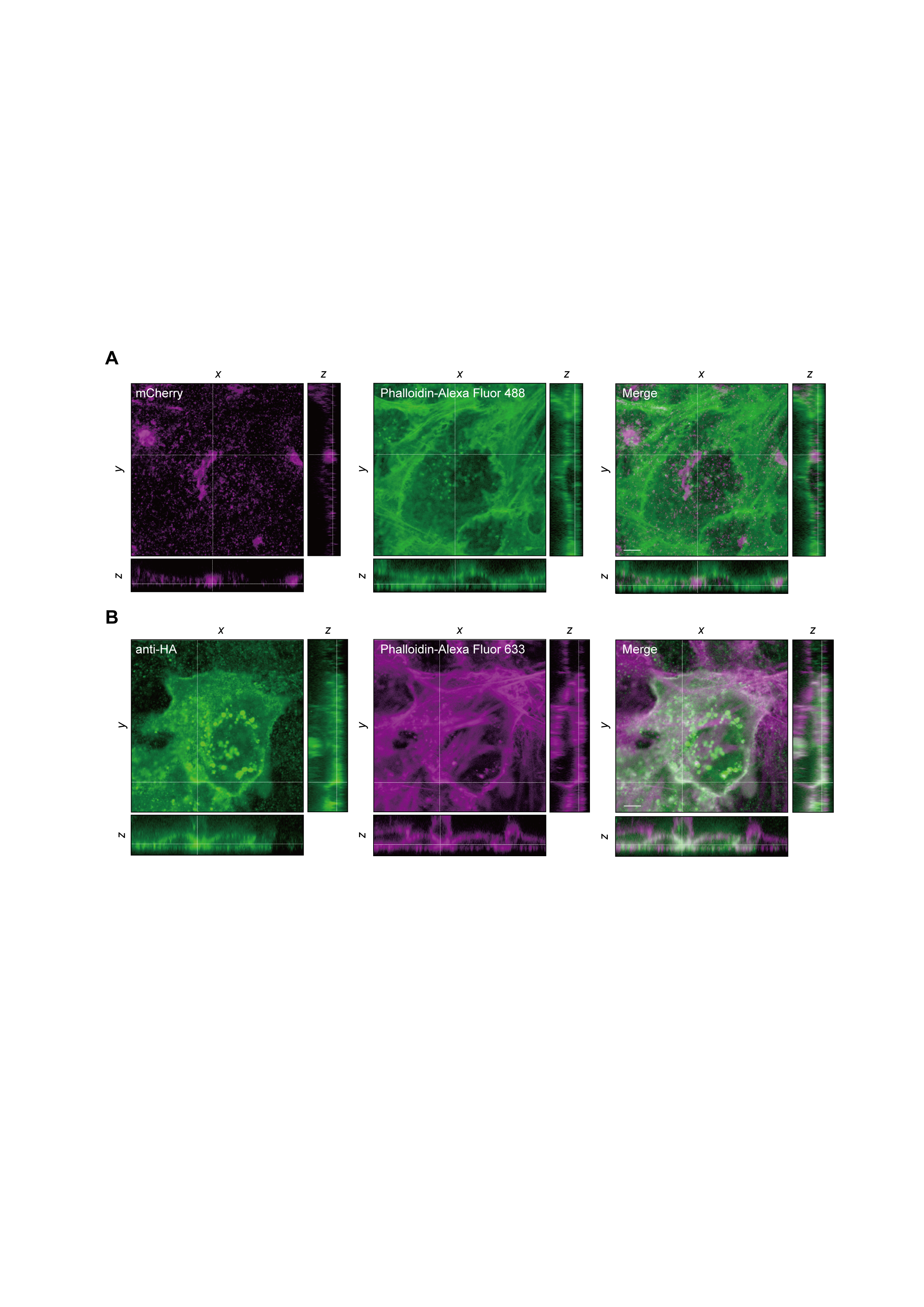

Supplement: S11 Fig — (A, B) IHGE cells were transiently transfected with a plasmid encoding Myc-mCherry–tagged HA-inserted JAM1 (magenta in A, green in B). Following 48 h of incubation, the cells were fixed, stained with Alexa Fluor 488–conjugated phalloidin (green) (A) or Alexa Fluor 633–conjugated phalloidin (magenta) (B), and then analyzed by immunofluorescence microscopy. Scale bars, 5 μm. (TIF) [file ppat.1008124.s011.tif]

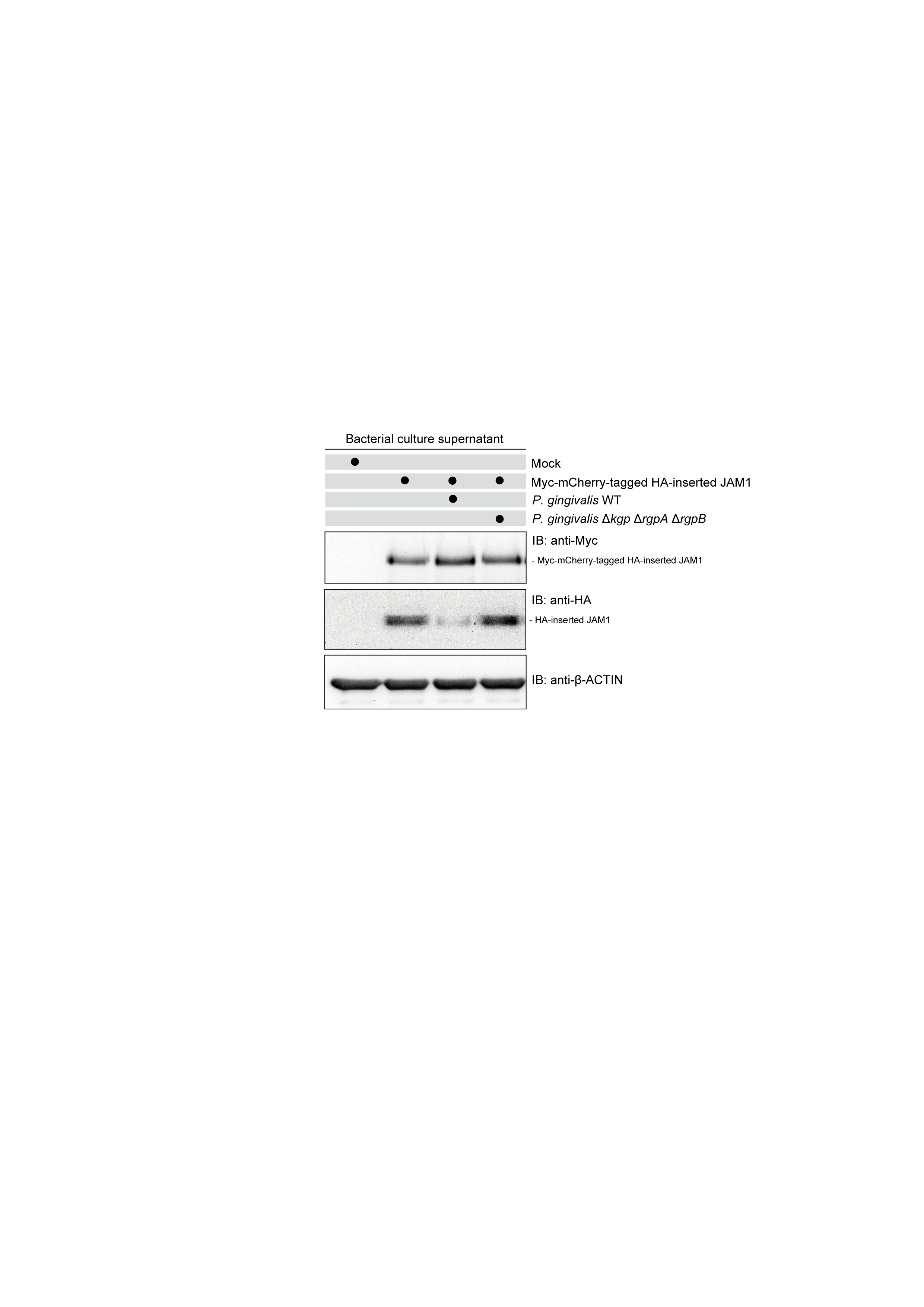

Supplement: S12 Fig — IHGE cells were transiently transfected with the plasmid of Myc-mCherry–tagged HA-inserted JAM1. Following 48 h of incubation, the bacterial culture supernatant of P. gingivalis WT or the Δkgp ΔrgpA ΔrgpB mutant was administered to IHGE cells for 1 h. The cells were then analyzed by immunoblotting with the indicated antibodies. (TIF) [file ppat.1008124.s012.tif]

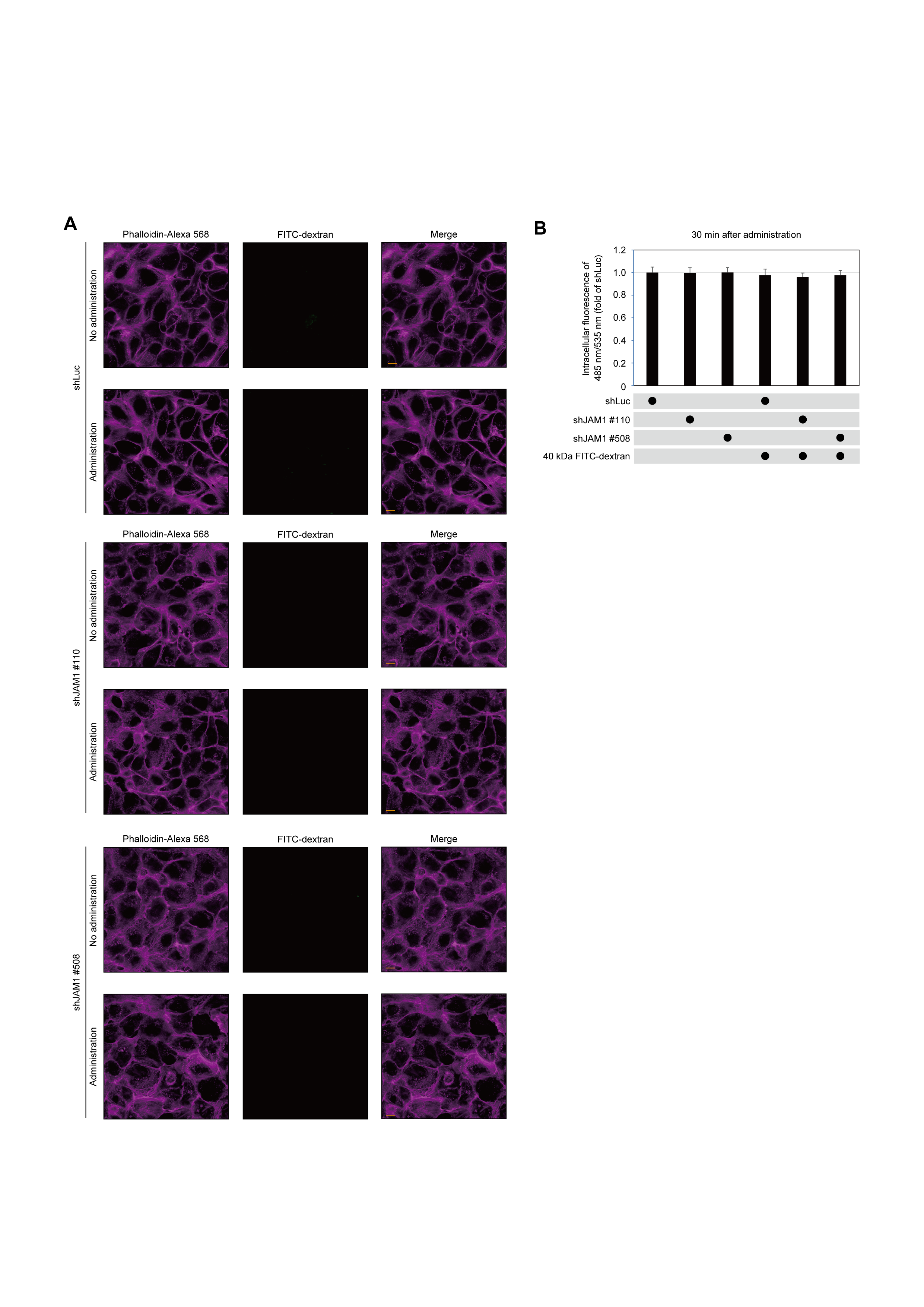

Supplement: S13 Fig — (A) IHGE cells stably expressing shLuc, shJAM1 #110, or shJAM1 #508 were treated with 40 kDa FITC–dextran (green) for 30 min. The cells were then fixed, stained with Alexa Fluor 568–conjugated phalloidin (magenta), and analyzed by confocal microscopy (without permeabilization). Bars, 10 μm. (B) Fluorescence (excitation: 485 nm, emission: 535 nm) in IHGE cells with or without administration of 40 kDa FITC–dextran for 30 min. Results are expressed as fold change relative to cells stably expressing shLuc without 40 kDa FITC-dextran and are the means ± SD of eight technical replicates. There was no statistically significant difference (p>0.05, two-tailed t test) between the presence and absence of 40 kDa FITC-dextran in any cell line. (TIF) [file ppat.1008124.s013.tif]

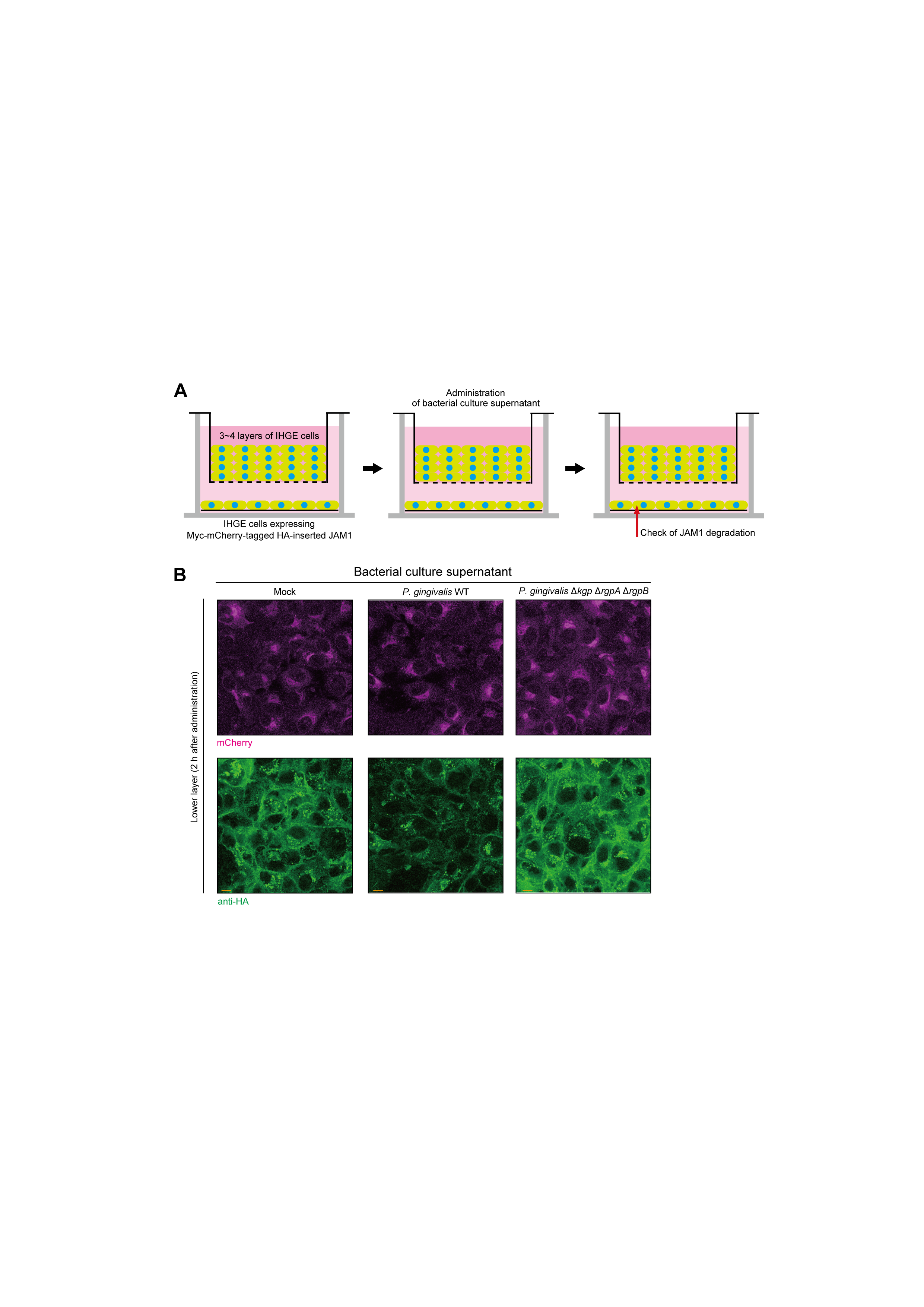

Supplement: S14 Fig — (A, B) Schematic image of the culture insert system (A). A multilayer of IHGE cells was cultured in the upper compartment, and a monolayer of IHGE cells expressing Myc-mCherry–tagged HA-inserted JAM1 was cultured on a coverslip in the lower compartment. Bacterial culture supernatant from P. gingivalis WT or the Δkgp ΔrgpA ΔrgpB mutant was administered to the tissues in the upper compartment. Following 2 h of incubation, the cells in the lower compartment were fixed, stained with anti-HA (green), and analyzed by confocal microscopy (B). Scale bars, 10 μm. (TIF) [file ppat.1008124.s014.tif]

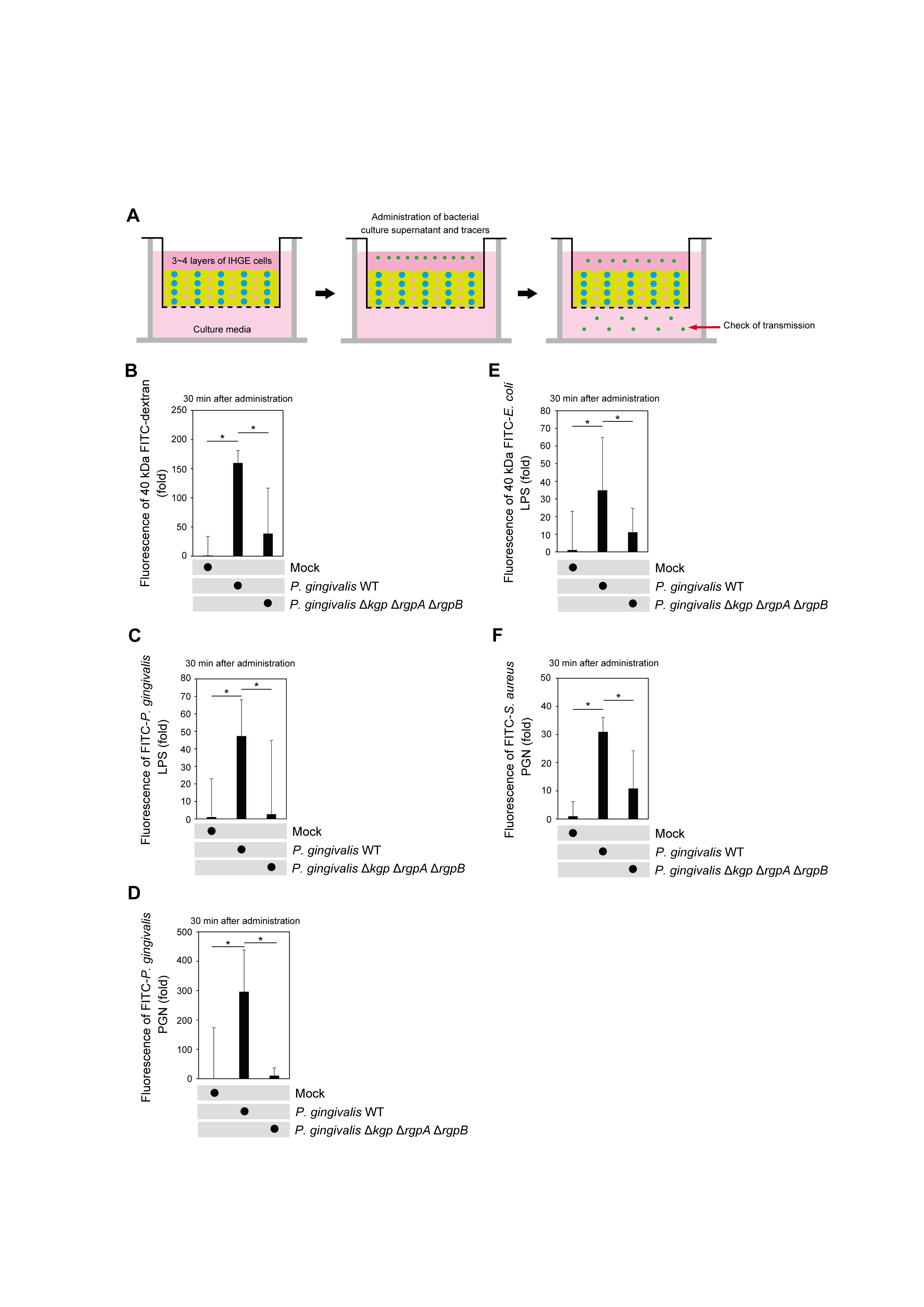

Supplement: S15 Fig — (A) Schematic illustration of 3D culture of IHGE cells. FITC tracer and bacterial culture supernatant from P. gingivalis WT or the Δkgp ΔrgpA ΔrgpB mutant were administered to the tissues in the upper compartment. Following 30 min of incubation, the transmission of tracer from the upper compartment to the lower compartment was analyzed by spectrometry. (B–F) Permeability to 40 kDa FITC–dextran (B), FITC–P. gingivalis LPS (C), FITC–P. gingivalis PGN (D), FITC–E. coli LPS (E), and FITC–S. aureus PGN (F) of gingival epithelial tissues treated with bacterial culture supernatant from P. gingivalis WT or the Δkgp ΔrgpA ΔrgpB mutant. Results are expressed as fold change relative to Mock and are the means ± SD of six technical replicates. *, p<0.05, one-tailed t test (closed testing procedure). (TIF) [file ppat.1008124.s015.tif]
