# Supplementary material for: Porphyromonas gingivalis induces penetration of lipopolysaccharide and peptidoglycan through the gingival epithelium via degradation of junctional adhesion molecule 1
Source: PLoS Pathog. 2019 Nov 7;15(11):e1008124. doi: 10.1371/journal.ppat.1008124 (PMC6932823; doi:10.1371/journal.ppat.1008124)

Figure 1A

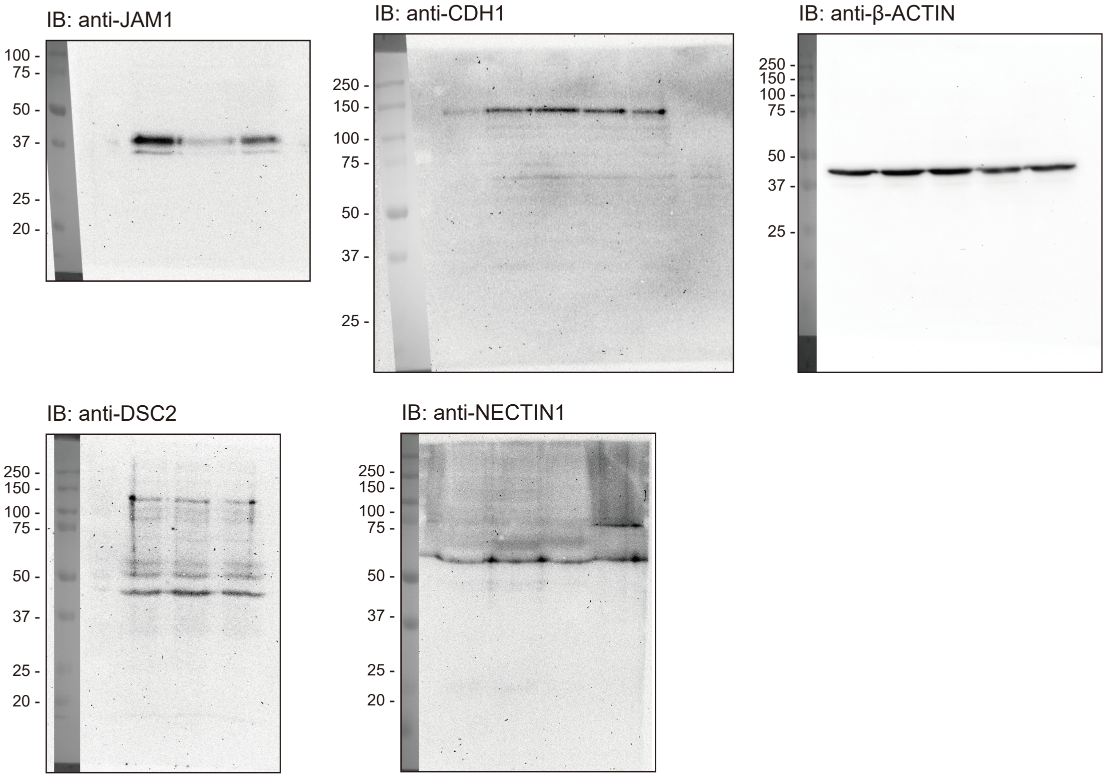

Figure 1B

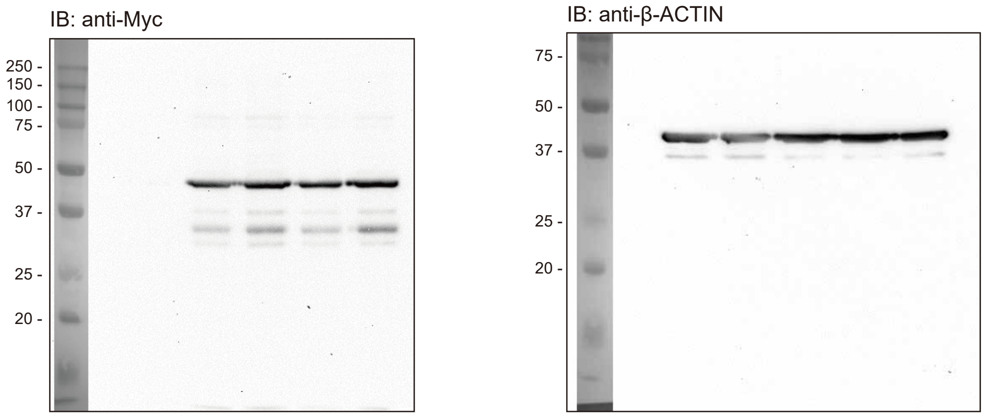

Figure 1C

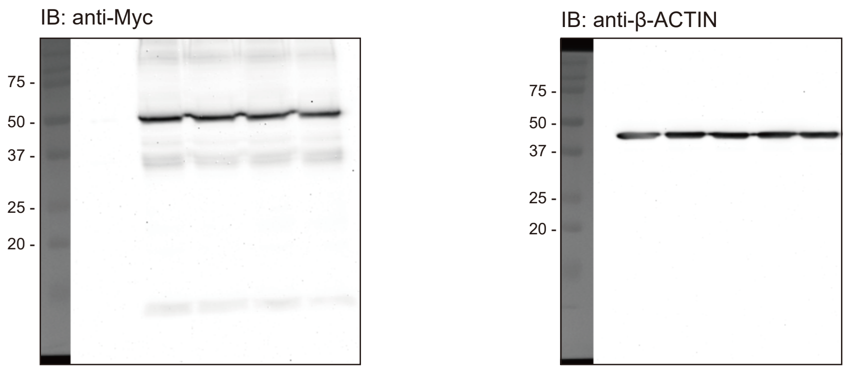

Figure 1D

IB: anti-Myc

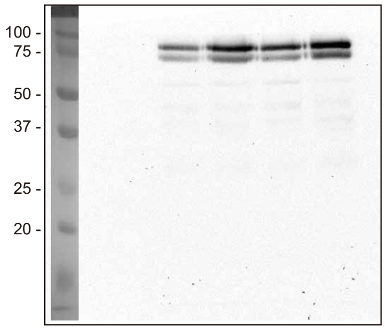

IB: anti-β-ACTIN

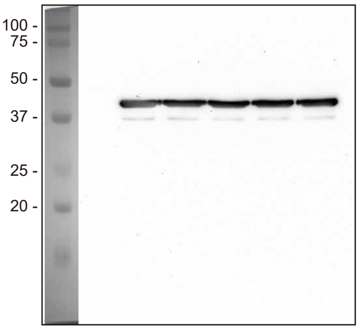

Figure 1E

IB: anti-HA

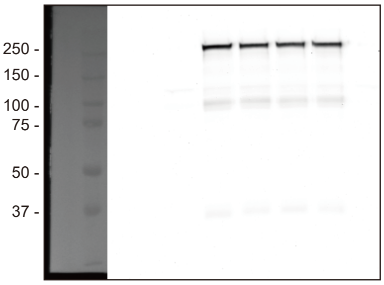

IB: anti-β-ACTIN

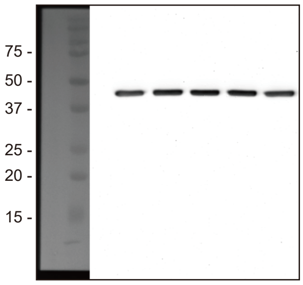

Figure 4B

IB: anti-HA

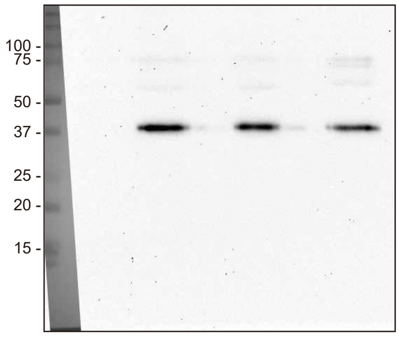

IB: anti-β-ACTIN

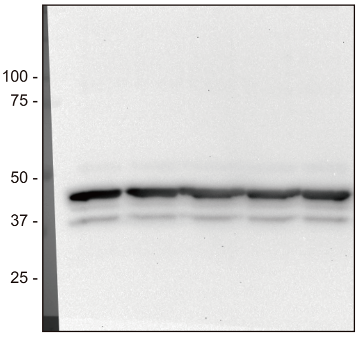

IB: anti-Myc

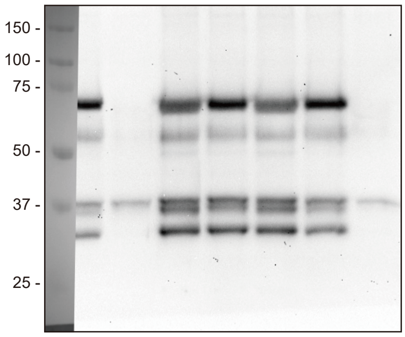

Figure 4C

IB: anti-HA

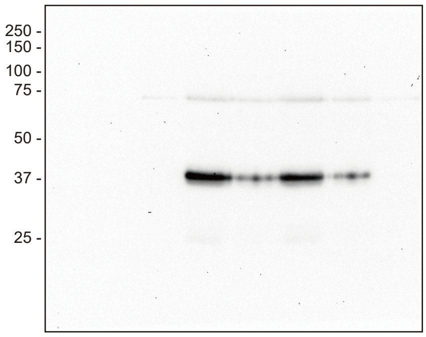

IB: anti-β-ACTIN

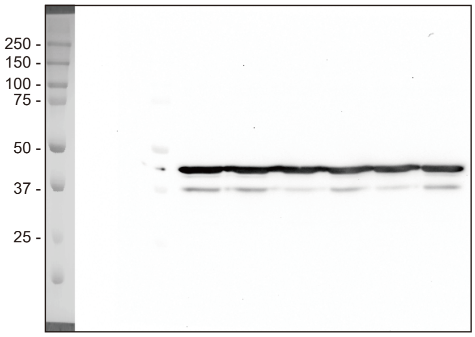

IB: anti-Myc

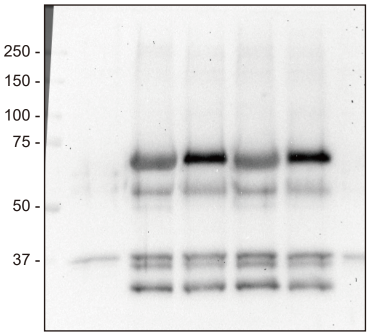

Figure 4D

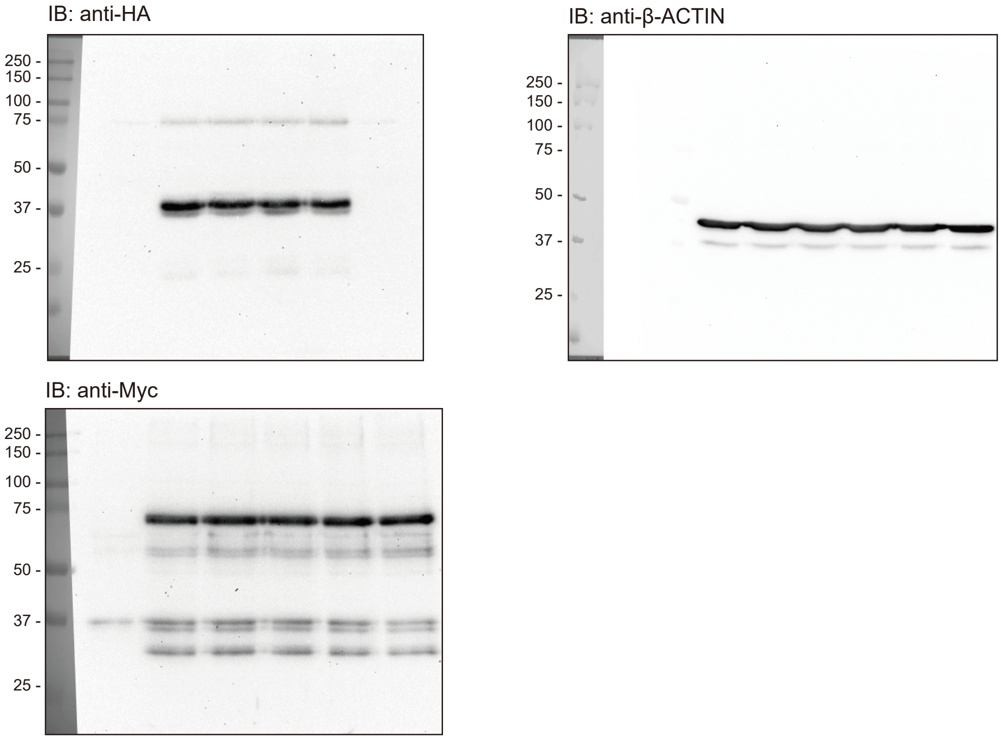

Figure 4E

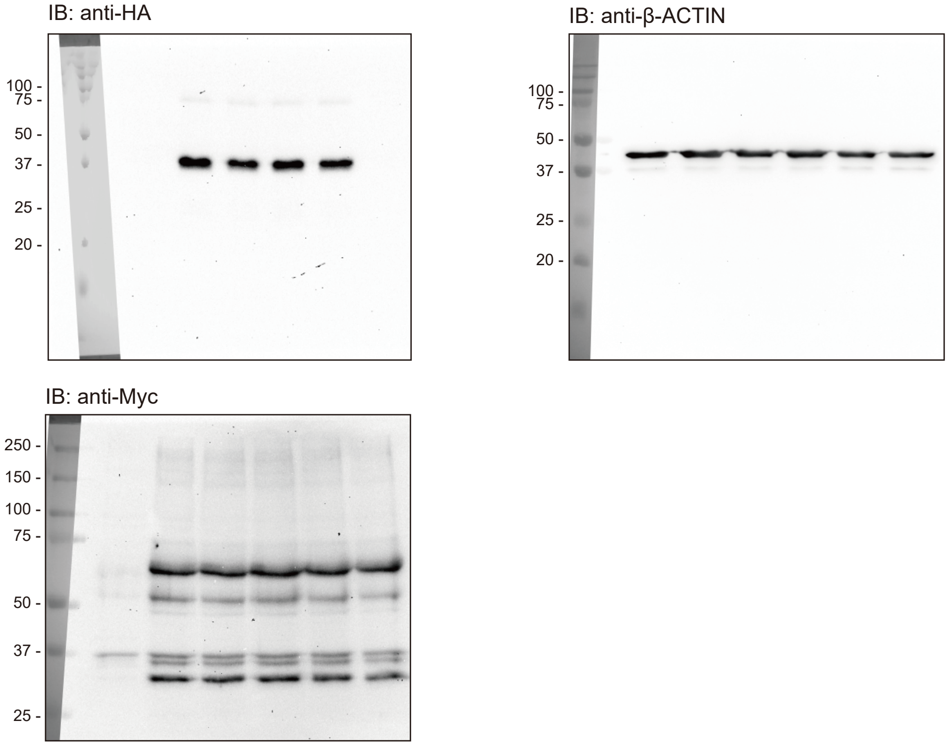

Figure 5B

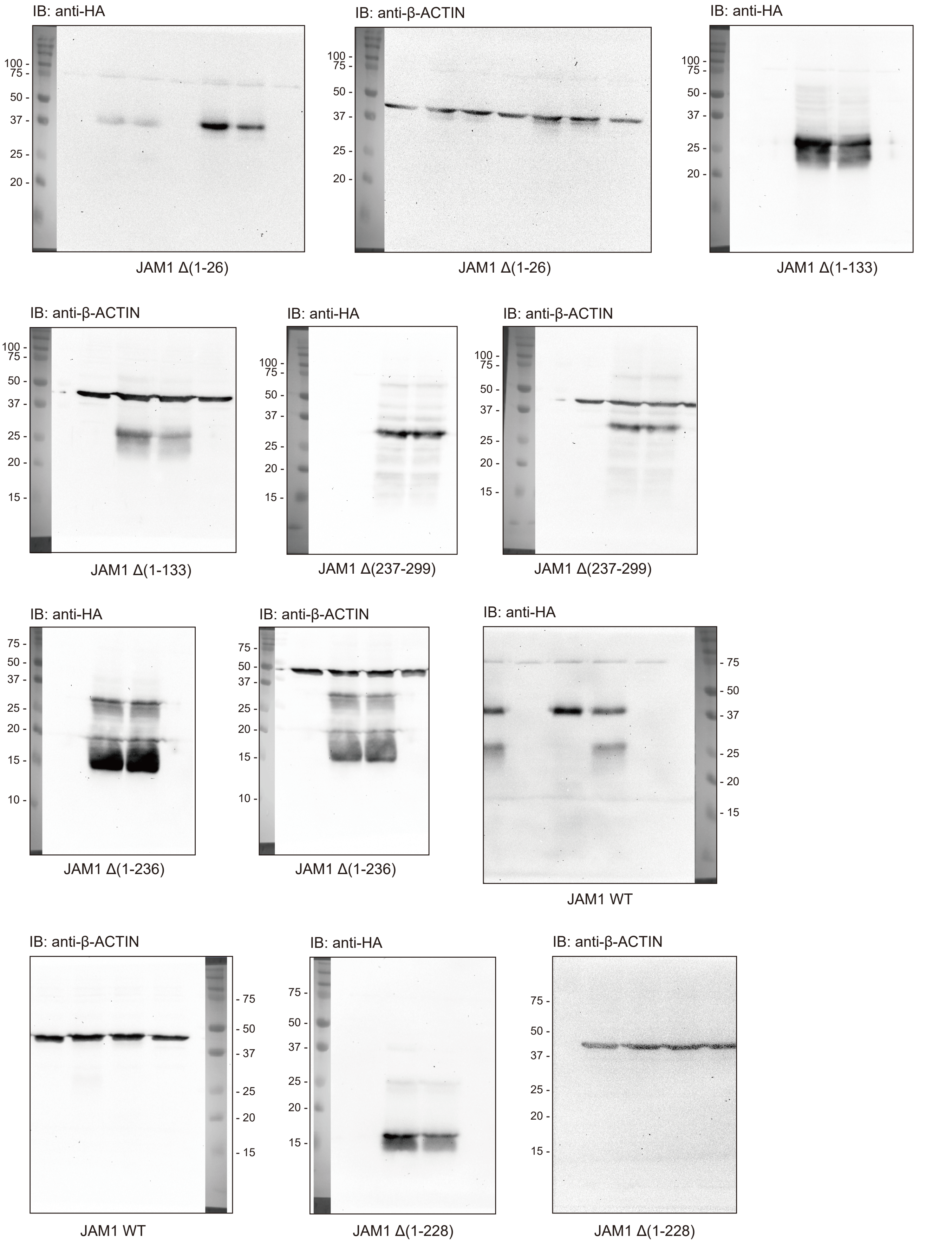
IB: anti-HA
100 - 75 - 50 - 37 - 25 - 20 -
JAM1 Δ(1-26)
IB: anti-β-ACTIN
100 - 75 - 50 - 37 - 25 - 20 -
JAM1 Δ(1-26)
IB: anti-HA
100 - 75 - 50 - 37 - 25 - 20 -
JAM1 Δ(1-133)
IB: anti-β-ACTIN
100 - 75 - 50 - 37 - 25 - 20 - 15 -
JAM1 Δ(1-133)
IB: anti-HA
100 - 75 - 50 - 37 - 25 - 20 - 15 -
JAM1 Δ(237-299)
IB: anti-β-ACTIN
100 - 75 - 50 - 37 - 25 - 20 - 15 -
JAM1 Δ(237-299)
IB: anti-HA
75 - 50 - 37 - 25 - 20 - 15 - 10 -
JAM1 Δ(1-236)
IB: anti-β-ACTIN
75 - 50 - 37 - 25 - 20 - 15 - 10 -
JAM1 Δ(1-236)
IB: anti-HA
- 75 - 50 - 37 - 25 - 20 - 15
JAM1 WT
IB: anti-β-ACTIN
- 75 - 50 - 37 - 25 - 20 - 15
JAM1 WT
IB: anti-HA
75 - 50 - 37 - 25 - 20 - 15 -
JAM1 Δ(1-228)
IB: anti-β-ACTIN
75 - 50 - 37 - 25 - 20 - 15 -
JAM1 Δ(1-228)

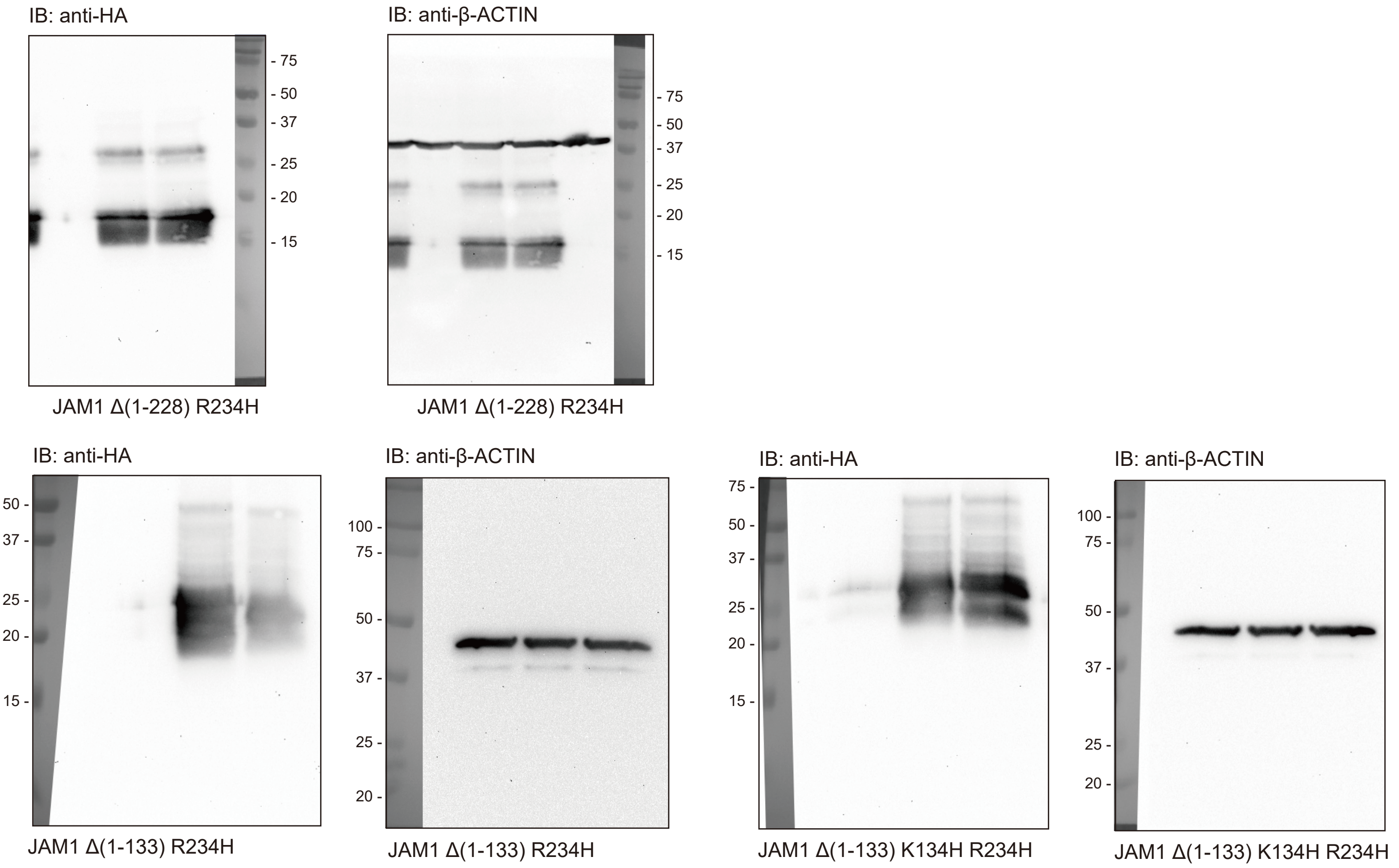

Figure 6B

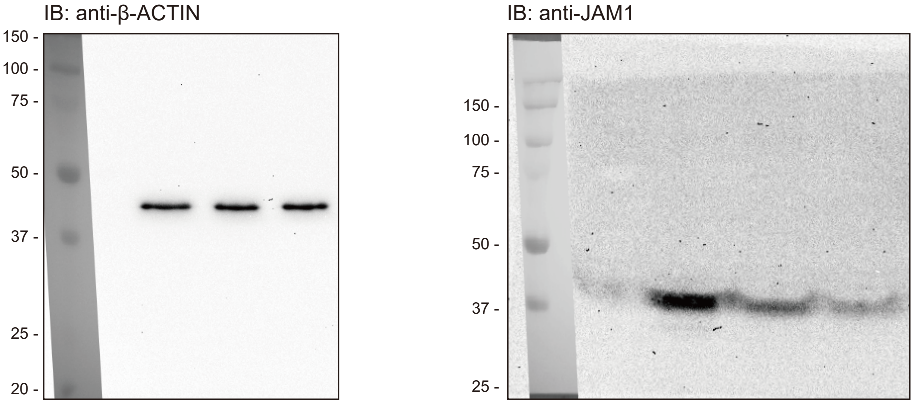

Figure 8H

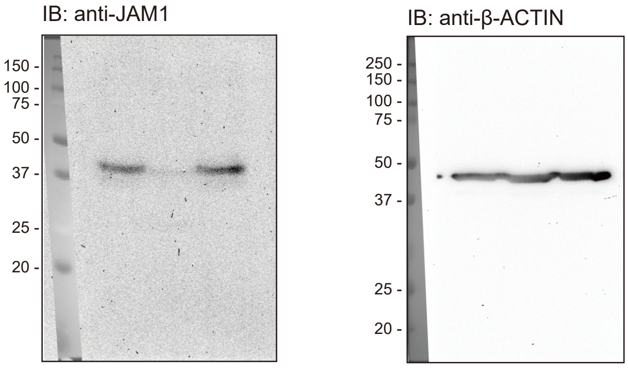

S1 Fig

IB: anti-JAM1

IB: anti-β-ACTIN

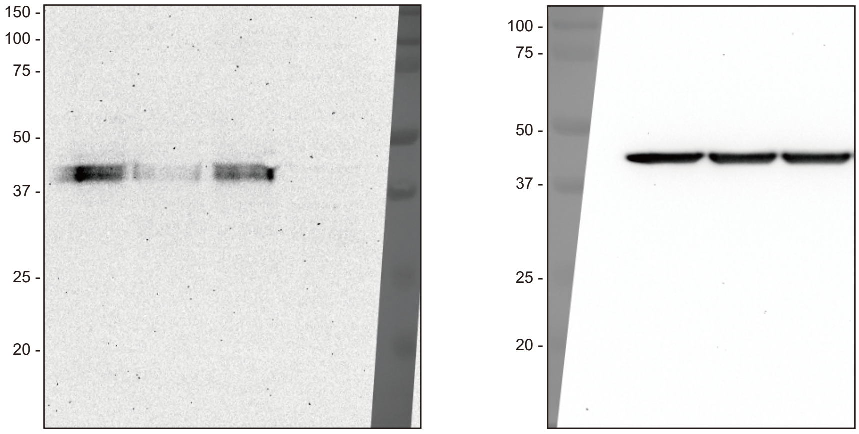

S2 Fig

IB: anti-Myc

IB: anti-β-ACTIN

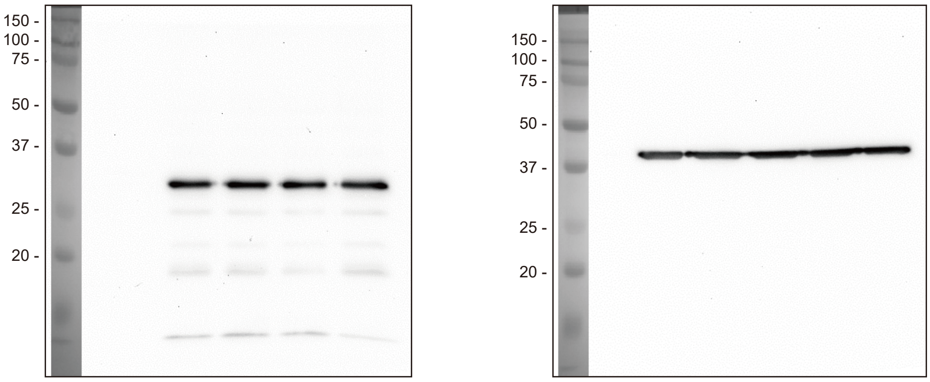

S7 Fig

IB: anti-Myc

IB: anti-HA

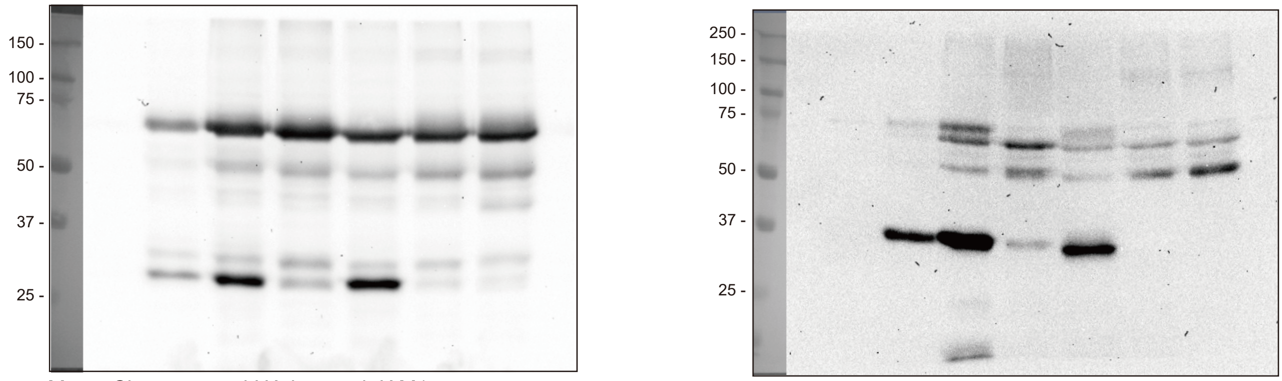

Myc-mCherry-tagged HA-inserted-JAM1

Myc-mCherry-tagged HA-inserted-JAM1

IB: anti-β-ACTIN

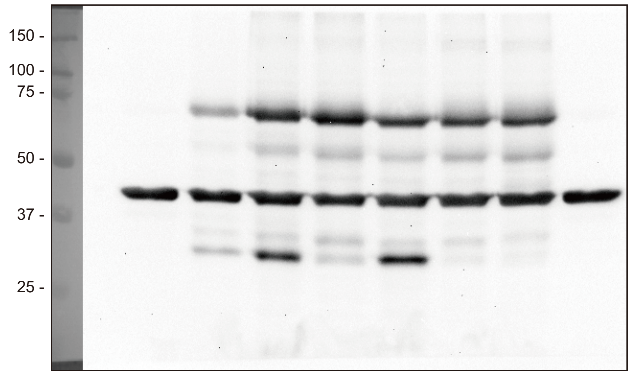

IB: anti-Myc

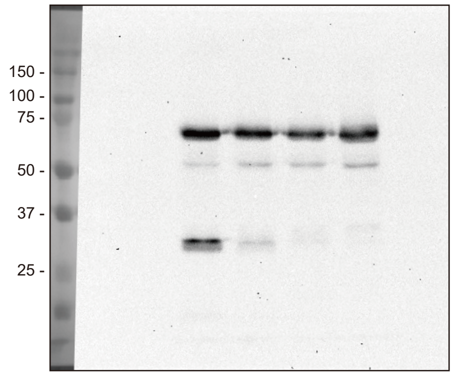

Myc-mCherry-tagged HA-inserted-JAM1

IB: anti-HA

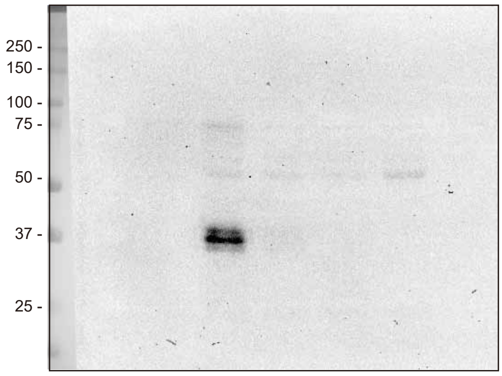

Myc-mCherry-tagged HA-inserted-JAM1

IB: anti-β-ACTIN

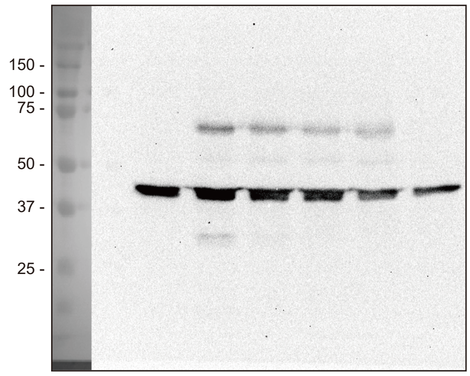

S12 Fig

IB: anti-Myc

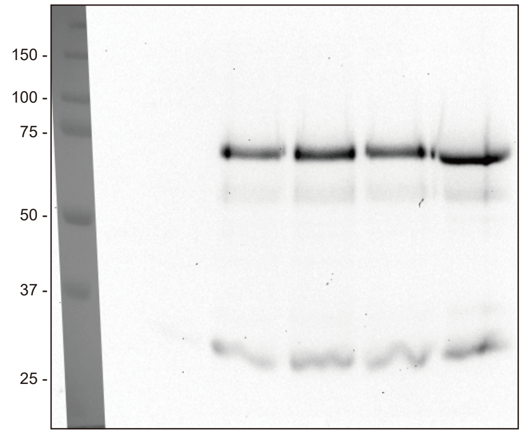

IB: anti-HA

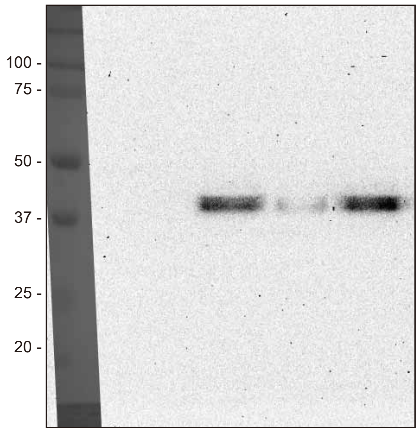

IB: anti-β-ACTIN

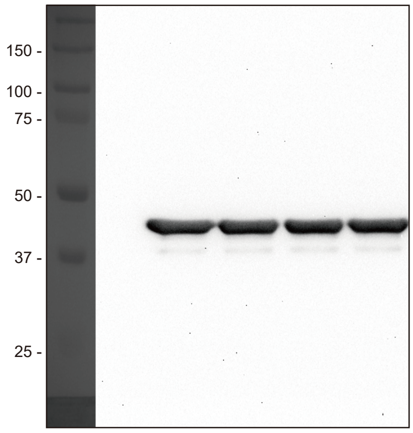

Supplement: S16 Fig — (PDF) [file ppat.1008124.s016.pdf]
